# In Silico Study Approach on a Series of 50 Polyphenolic Compounds in Plants; A Comparison on the Bioavailability and Bioactivity Data

**DOI:** 10.3390/molecules27041413

**Published:** 2022-02-19

**Authors:** Amalia Stefaniu, Lucia Camelia Pirvu

**Affiliations:** Department of Pharmaceutical Biotechnologies, National Institute for Chemical-Pharmaceutical Research and Development, ICCF, 112 Vitan Av., 031299 Bucharest, Romania

**Keywords:** plant phenolics, in silico study, oral bioavailability, bioactivity, GPCR ligand, ion channel modulator, kinase inhibitor, nuclear receptor ligand, protease inhibitor, enzyme inhibitor

## Abstract

Fifty (50) phytocompounds from several subclasses of polyphenols, chosen based on their abundance in the plant world, were analyzed through density functional methods, using computational tools to evaluate their oral availability and particular bioactivity on several cell modulators; key descriptors and molecular features related to the electron density and electrostatic potential for the lowest energy conformers of the investigated molecules were computed. An analysis of the bioactivity scores towards six cell modulators (GPCR ligand, ion channel modulator, kinase inhibitor, nuclear receptor ligand, protease inhibitor and enzyme inhibitor) was also achieved, in the context of investigating their potential side effects on the human digestive processes. Summarizing, computational results confirmed in vivo and in vitro data regarding the high bioavailability of soy isoflavones and better bioavailability of free aglycones in comparison with their esterified and glycosylated forms. However, by a computational approach analyzing Lipinski’s rule, apigenin and apigenin-7-*O*-rhamnoside, naringenin, hesperetin, genistein, daidzin, biochanin A and formonetin in the flavonoid series and all hydroxycinnamic acids and all hydroxybenzoic acids excepting ellagic acid were proved to have the best bioavailability data; rhamnoside derivatives, the predominant glycosides in green plants, which were reported to have the lowest bioavailability values by in vivo studies, were revealed to have the best bioavailability data among the studied flavonoids in the computational approach. Results of in silico screening on the phenolic derivatives series also revealed their real inhibitory potency on the six parameters studied, showing a remarkable similitude between the flavonoid series, while flavonoids were more powerful natural cell modulators than the phenyl carboxylic acids tested. Thus, it can be concluded that there is a need for supplementation with digestive enzymes, mainly in the case of individuals with low digestive efficiency, to obtain the best health benefits of polyphenols in humans.

## 1. Introduction

In the context of the undeniable evidence of the efficacy of plant compounds (particularly of secondary metabolites), both in the prevention and treatment of human diseases [1,2,3,4,5,6,7], a reasonable question is whether the plant-based products with a high content of specific active compounds (e.g., products enriched in so-called natural antioxidants) could also lead to unwanted side effects. Rationally, the most affected biological processes should be at the level of the digestive system, with which they come into direct contact. Further, depending on their metabolism and bioavailability in humans, beneficial or less beneficial effects of the secondary metabolites from plants are expected in the case of digestive processes, as well as systemically. The most likely side effects are expected in the case of plant-based products used for handling chronic conditions, such as those related to chronic inflammation (e.g., arthritis and inflammatory bowel diseases) [8,9,10] and metabolic diseases (e.g., diabetes and obesity) [11,12,13], that are essentially based on antioxidant and anti-inflammatory compounds (polyphenols and other active classes) to reduce the inflammation process and sugar absorption in humans. Their potential, harmful effects could primarily occur due to the inhibitory activity upon the digestive enzymes, leading to the incomplete digestion of carbohydrates, proteins and lipids, which, further, could cause the initiation or deepening of food intolerance, irritable bowel syndrome and other bowel diseases. Furthermore, the most prominent secondary metabolites in plants, polyphenolic compounds, are effective antimicrobial agents; therefore, the sustained ingestion of plant products and selective phytochemicals ascribed to antimicrobial properties could affect the human intestinal bacterial community, the microbiota.

The inhibitory activity of polyphenol compounds on the alpha-glucosidases’ activity is a well-known subject, considered for several decades [14,15]. Thus, in vivo clinical studies on healthy male volunteers (none with symptoms or history of gastrointestinal/GI disease) receiving placebo, acarbose and miglitol (two common alpha-glucosidase inhibitors) concluded that “alpha-glucosidase inhibitors accelerate mouth to caecum transit time by inducing carbohydrate malabsorption” [15]. The main benefit and application of this inhibitory activity is the successful control of postprandial hyperglycemia in diabetics [16,17]. The mechanism of action of plant polyphenols primarily consists of the inhibition of two glycoside hydrolases, alpha-glucosidase and alpha-amylase [18,19], which transform soluble polysaccharides from food into glucose molecules. The second, and more effective, anti-diabetes mechanism consists of the capacity of plant polyphenols to modulate the function of glucose receptor and the expression of glucose transporter (GLUT2) in pancreatic beta cells producing insulin in humans [16,17].

Likewise, in the 1980s, the ability of plant polyphenols to inhibit in vitro activity of all the main digestive enzymes in humans (e.g., typsin, alpha-amylase and lipase) was proven, and flavan derivatives, such as anthocyanins, ellagitannins and condensed tannins, were established as the most potent plant inhibitors (namely antinutritional factors) [14]. For example, the polyphenol fraction from species of berries (known to contain the highest levels of anthocyanin, procyanidin and catechin derivatives in plants) was proved to inhibit both carbohydrate-degrading enzymes (the extracts from blueberry and blackcurrant were the most effective inhibitors of alpha-glucosidase enzymes) and lipase activity in humans [18]. Further studies on 30 crude extracts from common fruits confirmed the augmented inhibitory activity of catechin-rich fruits. For example, while blue honeysuckle and red gooseberry fruits were shown to induce the highest inhibitory activity on carbohydrate-degrading enzymes, the lingonberry fruit extracts indicated the strongest anti-lipase activity; specifically, in the case of alpha-glucosidase, the measured IC_50_ values were between 39.91 and 400 mg/mL and, in the case of alpha-amylase, between 1.04 and 80 mg/mL, while, in the case of lipase activity, there were counted values from 0.72 to 135.07 mg/mL [20]. A relatively low value of IC_50_ in the case of alpha-amylase and lipase is an indication of the potency of plant products with a high content of flavan derivatives in inhibiting the activity of the main digestive enzymes in humans.

The usefulness of plant polyphenolic compounds for human beings and health issues is undeniable [21,22] but, in the context of evidence of their inhibitory activity on glucosidases, proteases and lipase, in conjunction with their antimicrobial properties, their negative potential on human health should also be considered, especially in the context of the inhibition of the main digestive enzymes in humans, as well as the inhibition of the microbial community in the intestine. Regarding the capacity of the human body to counteract the potential negative effects of polyphenolic compounds upon the digestive processes, both by enzyme inhibition and disturbing the microbiota, it is believed that the human body is able to further synthesize the inhibited enzymes [23] as some polyphenolic compounds also have prebiotic potency [24]. However, these processes are deficient in the elderly and in the case of susceptible individuals. In addition, it is considered that a complex microbiota is more resistant to the potential negative influence of plant polyphenols [25], but it is also stated that there are no means to predict whether the consequences of a diet supplemented with high doses of phytocompounds will be beneficial or will be harmful. This tends to depend on the individual gut sensibility and specific microbiota of a person. In the case of susceptible individuals, the inhibition of the digestive enzymes could overlap the antimicrobial effects of the plant compounds [25,26,27], together encouraging food intolerance, intestinal inflammation, malabsorption and microbial impairments in the intestine, namely dysbiosis. Studying the relationship between dysbiosis and the inhibition of the digestive enzymes, specifically by measuring the digestive value of the microbial flora in dogs [28,29,30], the evaluation of the predominant bacterial gene categories in the canine gut indicated the following results: 12–13% of all sequences were involved in carbohydrate metabolism, 7–9% in protein and amino acid metabolism, 7–8% in cell wall synthesis, 6% in vitamin and cofactor synthesis and 7% in nucleic acid synthesis [28]; these data are in the context of studies on the microbial flora, which revealed functional and disease similarity between humans and small animals, cat, dogs and mice, respectively [31].

Defined as a decrease in the diversity of the microbiome or changes in the relative proportion of certain organisms in the intestine, with or without the presence of a pathogenic flora [32], dysbiosis is related to many gastrointestinal and even systemic diseases, particularly those with inflammation of the intestinal mucosa [33,34]. Studies revealed that the common constituents of the microbiota in humans and small animals are *Firmicutes*, *Bacteroidetes*, *Proteobacteria*, *Fusobacteria* and *Actinobacteria*, the dysbiosis process in mice being characterized by an increased *Bacteroidetes* ratio in intestine [33]; dysbiosis in cats was associated with a decreased number of total bacterial species, particularly *Bacteroides* spp. and *Bifidobacterium* spp. and, at the same time, an increased ratio of *Desulfovibrio vulgaris*, a sulfate-reducing bacterial group capable of producing hydrogen sulfides. The presence of *Desulfovibrio* spp. was associated with bacteremia [35,36] and inflammatory bowel disease for felines [37] but also in humans [38].

Moreover, studies revealed that some of the most notorious, anti-inflammatory plant compounds, β-boswellic acid and its derivatives (acetyl-11-keto-β-boswellic acid, acetyl-11-keto-α-boswellic acid and acetyl-β-boswellic acid) and also betulinic acid, act as selective ciclooxigenase-1 (COX-1) inhibitors, while phenethyl-*trans*-ferulate and ruburic acid act as non-selective COX-1 (anti-inflammatory enzyme) and COX-2 (pro-inflammatory enzyme) inhibitors [39], meaning potential negative effects upon the digestive system in patients treated for chronic, inflammatory diseases [40].

In summary, due to the difficulty and the complexity of in vivo studies on small animals and, even more so, of clinical trials in humans, the computational analysis on the most common polyphenolic compounds to reveal their ability to impact the activity of some essential cell modulators (e.g., receptors, enzymes and ion channels) could give some information on their potential negative influence, particularly on the biological processes at the level of digestive system in humans. Accordingly, the aim of the present work was to perform an in silico approach on the interactions of fifty (50) polyphenol compounds in plants with some of the most important cell modulators (e.g., G-protein-coupled receptors/GPCR, ion channel modulators, kinase inhibitor, nuclear receptor, protease inhibitor and enzyme inhibitor) for the purpose of analyzing their potential harmful effects in relation to the digestive processes in humans; the fifty plant compounds belonged to ten sub(sub)classes of polyphenols, flavonoid and phenylcarboxylic acid derivatives, and they were selected based on their frequency and usage in food products, herbal medicines, food and dietary supplements, nutricosmetics and other plant-based products. Specifically, 12 flavone derivatives (apigenin and luteolin derivatives), 14 flavonol derivatives (kaempferol, quercetin and myricetin derivatives), 4 flavanone derivatives (naringenin and hesperitin derivatives), 2 flavan derivatives (catechin and epicatechin), 4 isoflavones (genistein, daidzin, formonetin and biochanin A), 10 hydroxycinnamic acid derivatives (coumaric, ferulic and caffeic acid derivatives) and 4 hydroxybenzoic acid derivatives (gallic acid, ellagic acid, salicylic acid and vanillic acid) were analyzed. The studies were carried out in the context of an analysis of bioavailability of the 50 plant compounds in comparison with in vitro and in vivo results from a systematic review of the literature.

## 2. Results

### 2.1. Computed Molecular Properties

The first part of the study was intended to predict molecular properties for the fifty (50) plant compounds grouped as: quercetin derivatives (Table 1), kaempferol derivatives (Table 2), myricetin derivatives (Table 3), luteolin derivatives (Table 4), apigenin derivatives (Table 5), flavanone and flavan derivatives (Table 6), isoflavone derivatives (Table 7), hydroxycinnamic acid derivatives (Table 8) and hydroxybenzoic acid derivatives (Table 9); their chemical formulas (as optimized structures) are available in the supplementary material, Figures S1–S9. Further, the optimized structures, molecular properties and chemical-physical features of the fifty test compounds were obtained [41,42,43,44]: e.g., area, volume, polar surface area (PSA and TPSA), ovality, polarizability, dipole moment, water–octanol partition coefficient (log*P* and milog*P*), one descriptor related to the flexibility of molecules and the number of rotatable bonds (nrotb), respectively, as well as the RO5 (rule of the five) parameter following Lipinski’s criteria for estimating the feasible oral bioactivity of a drug/compound [45,46].

Given the high share of oral bioavailability of a compound, the results of DFT calculations (Table 1, Table 2, Table 3, Table 4, Table 5, Table 6, Table 7, Table 8 and Table 9) were plotted as milog*P* along nrotb, as shown in Figure 1, Figure 2, Figure 3, Figure 4, Figure 5, Figure 6, Figure 7, Figure 8 and Figure 9.

As expected, area (A) and volume (V) of all the series of tested compounds varied in the same trend as the molecular weight (MW), while polar surface area (PSA) stood out independently, depending on the number and configuration of hydroxyl groups in the specific compound. PSA values were due to the van der Waals surface of all nitrogen and oxygen atoms and any hydrogens attached to these electronegative atoms. PSA reflected the presence of oxygen sp^3^ or sp^2^ and increased mainly with the increase of hydroxyl groups, which are very abundant in polyphenols and their glycosides, but also carbonyl and methoxy groups, and their disposal on the molecule’s skeleton; it can be noticed that hydrogen and hydroxyl ions are directly responsible for the chemical and physical stability and degradation of an active compound (e.g., the active ingredient in a drug), being involved in specific acid-base catalysis [47], and are considered during preformulation step assays of drug candidates. In addition, the PSA value is important when estimating the rate at which molecules can go through hydrophilic or hydrophobic media; therefore, it influences the bioavailability of a compound. In this context, correlative to the fifty plant polyphenols studied, most indicated a predictable PSA dynamic; the PSA value rose from flavonol aglycone to flavonol (poly)glycoside and showed an increasing magnitude from the 3-*O*-rhamnoside to 3-*O*-glucoside and 3-*O*-galactoside derivatives. Flavone derivatives were less predictable concerning the PSA dynamic, and luteolin derivatives performed, once again, a particular behavior compared to other flavonoid derivatives, also noticed in the case of antioxidant activity, i.e., resistance to hydrolysis and high difficulty in the process of chemical derivatization. Flavanones and isoflavones also showed an increasing magnitude with an increasing number of hydroxyl groups. In the specific case of phenolic acids, the PSA value also rose from aglycone to its esterified/condensed forms and showed an increasing magnitude with the increasing number of hydroxyl groups. In terms of TPSA (predicted with Molinspiration), a similar dynamic with PSA was observed, and the same behavior was noticed for luteolin derivatives. TPSA represents the topological surface area calculated as a sum of the fragment-based contribution. The available surface area influences the intermolecular contact; therefore, the compact shape of the molecules provides a smaller available surface area for intermolecular interactions and weaker dispersion forces. Interactions with amino acid residues of the target proteins occur within the accessible area of ligands too.

The ovality index is a more complex molecular descriptor associated with the effective molecular shape of molecules obtained as area, volume and PSA from the three-dimensional, space-filling model; it represents the deviation from spherical shape, where 1.0 corresponds to spherical top molecules and values greater than (>) 1.0 indicate deviation from the sphere. Generally, the values of ovality index increase with increasing linearity of the molecule. In the present study, the smallest deviations from the spherical shape were shown by quercetin (1.40), kaempferol (1.38), myricetin (1.41), luteolin (1.38), apigenin (1.37), naringenin (1.4), biochanin A (1.40), quinic acid (1.28), p-coumaric acid (1.29) and salicylic acid (1.21). These data were added to another computer-assisted drug design (CADD) study of 16 flavonoids compounds which concluded that 5,7-dihydroxy flavonoid compounds were the best trypsin and trypsin-like enzyme inhibitors; quercetin, myricetin and morin had the best structural configuration due to their suitably located hydroxyl groups and their planar configuration [47].

Complementary predictive data, for better understanding of the electronic structure of compounds, are given by dipole moment and polarizability (alpha polarizability parameter), related to aspects of the electronic, vibrational structure and bonding [48]. The logarithm of the water–1-octanol partition coefficient (Log*P*) of compounds, an indication of lipophilicity, is used in the pharma field to predict properties and transport behavior of molecules. Water mimics the aqueous cellular or extracellular media, while 1-octanol is employed as an organic model for the lipid biological membrane; yet this organic solvent has some limitations due to the presence of its free hydroxyl group, water inclusion (4%, *v/v*) and hydrogen bonding capability. Its use can attenuate the crossing membrane ability for compounds able to form H bonds and considered to have high hydration potential. However, Log*P* is an accessible and widely used descriptor for predicting lipophilicity in pharma screenings. In Spartan software, Log*P* values are estimated by employing the widely used atomic contribution method of Ghose, Pritchett and Crippen [49]; developed by a Molinspiration methodology, miLog*P* is a sum of fragment-based contributions and correction factors. Finally, a zero or single violation of Lipinski’s criteria (RO5 parameter) is assigned to feasible, orally active drugs [50].

Thereby, the computational analysis revealed that 33 of the 50 phytocompounds tested were in good agreement with Lipinski’s rule. Generally, the lead compounds (aglycones) of each flavonoid series had no Lipinski’s violation: e.g., Q, K, L and M (1 violation). Interestingly, apigenin derivatives (A) were all feasible compounds excepting the rutinoside derivative; non-substituted flavanones (N, H), as well as isoflavones and all flavan and hydroxycinnamic acid derivatives, showed zero or one Lipinski’s violations. However, the molecules that remained feasible following the double analysis, RO5 and log*P*, were as follows: apigenin and apigenin-7-*O*-rhamnoside, naringenin, hesperetin, genistein, daidzin, biochanin A and formonetin in the flavonoid series and all hydroxycinnamic acids and all hydroxybenzoic acids excepting the condensed form of ferulic acid, namely ellagic acid. Veber’s rule [51] supplements Lipinski’s filter by introducing limitation to polar surface area (PSA) values (no larger than 140 Å^2^) and to the number of rotatable bonds (recommended less than 10) for good oral bioavailability. Furthermore, the flexibility of a molecule (estimated by the nrotb parameter) plays an important role in establishing interactions within the amino acids from the active binding site of the enzyme; no rotatable bond on the structure indicates rigid molecules. Relative to the fifty test compounds, the computational analysis indicated that the more flexible compounds were those containing rutinoside residues (nrotb = 6), glucoside and galactoside residues (nrotb = 4) and rhamnoside residue (nrotb = 3); curcumin (nrotb = 8), rosmarinic acid (nrotb = 7), chlorogenic acid and its isomers (nrotb = 5) and sinapic acid (nrotb = 4) were also flexible molecules. The number of rotatable bonds and molecular flexibility also provide clues as to whether the compound crystallizes or not [52]; increased flexibility means a lower tendency to crystallization [53,54]. Furthermore, acknowledged as a measure of the hydrophilicity of an orally administered compound and, at the same time, a measure of the molecule flexibility, milog*P* analysis in correlation with nrotb generates a bioavailability scale for a series of compounds studied. From a computational point of view “reduced molecular flexibility, as measured by the number of rotatable bonds, and low polar surface area or total hydrogen bond count (sum of donors and acceptors) are found to be important predictors of good oral bioavailability, independent of molecular weight” [51]. In addition, a planar conformation of a molecule results in a better interaction with serum albumin and, therefore, in a better bioavailability in humans [55].

**Figure 1:**
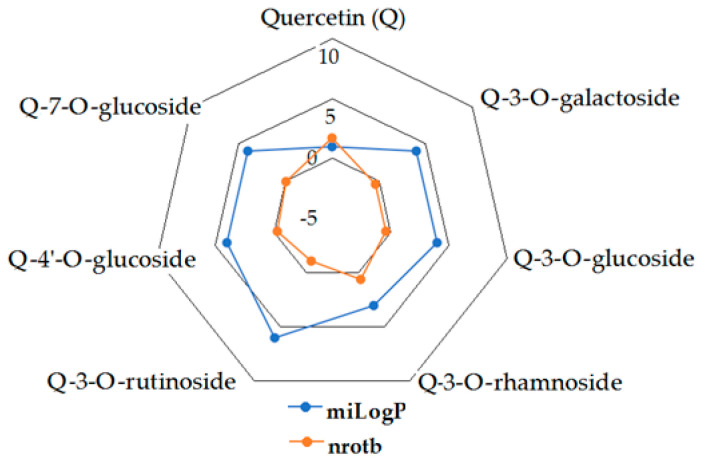
MiLog*P* along with nrotb for Q derivatives.

**Figure 2:**
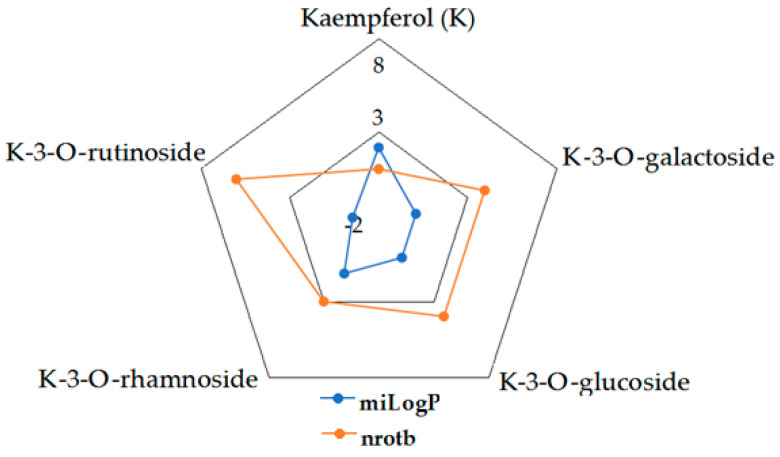
MiLog*P* along with nrotb for K derivatives.

**Figure 3:**
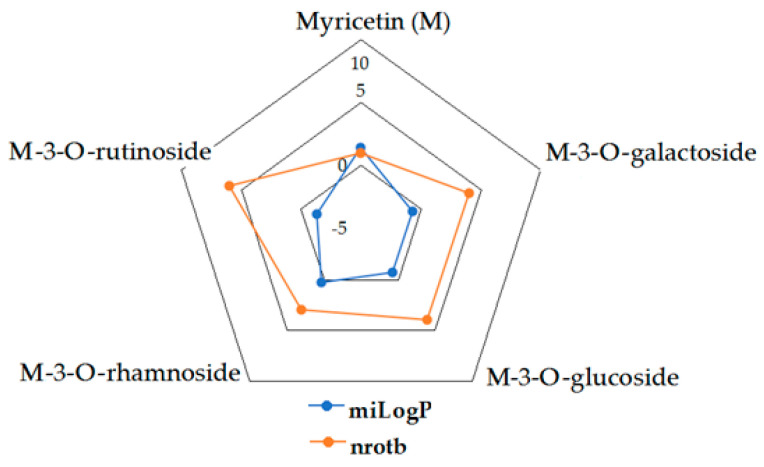
MiLog*P* along with nrotb for M derivatives.

**Figure 4:**
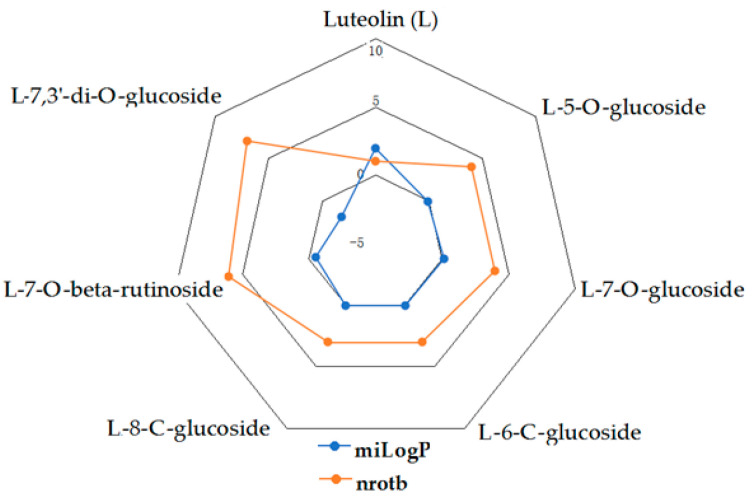
MiLog*P* along with nrotb for L derivatives.

**Figure 5:**
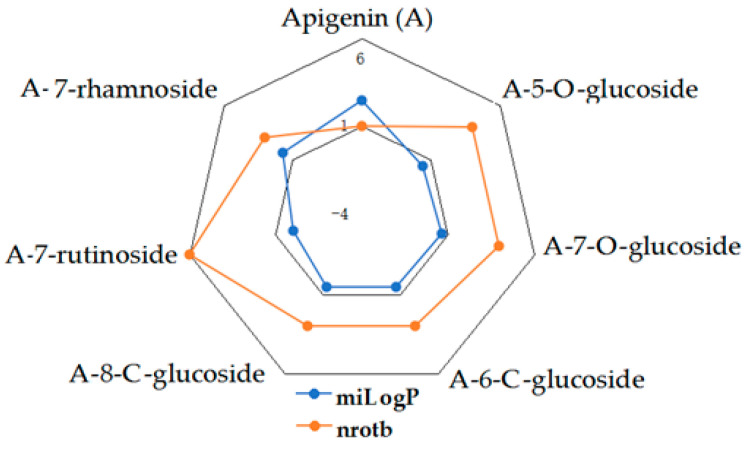
MiLog*P* along with nrotb for A derivatives.

**Figure 6:**
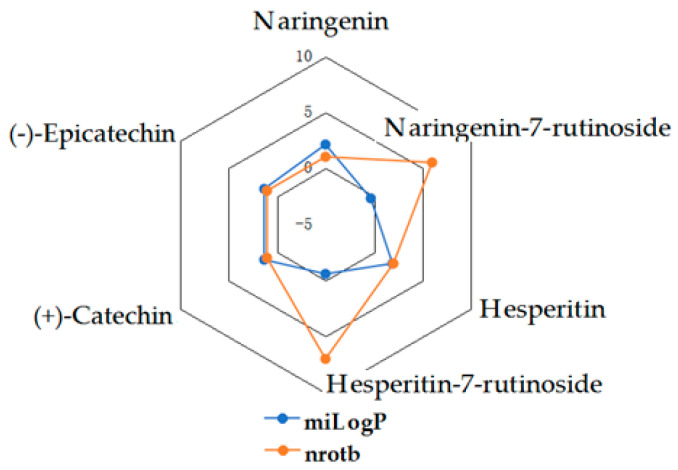
MiLog*P* along with nrotb for flavanone and flavan derivatives.

**Figure 7:**
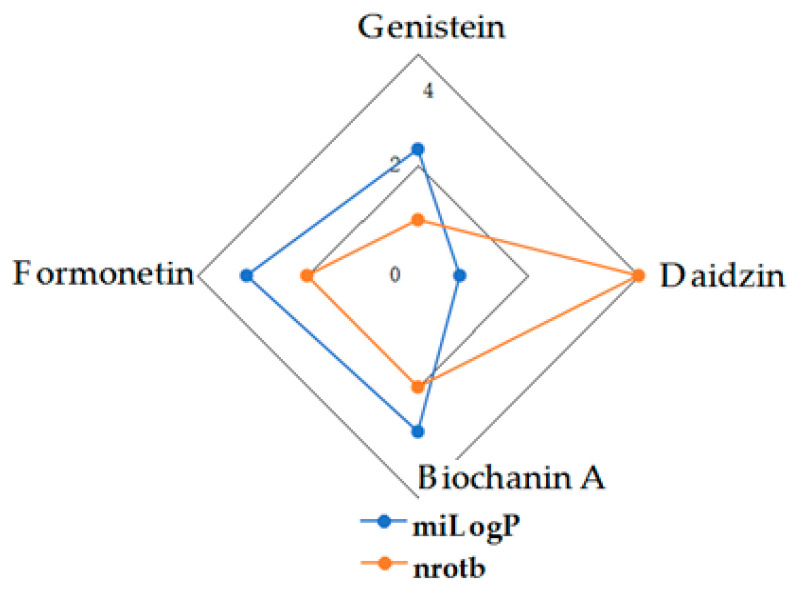
MiLog*P* along with nrotb for isoflavone derivatives.

**Figure 8:**
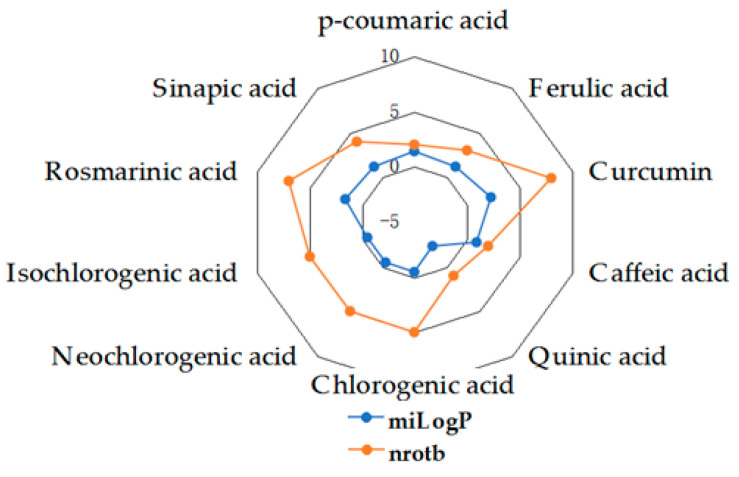
MiLog*P* along with nrotb for HCAc derivatives.

**Figure 9:**
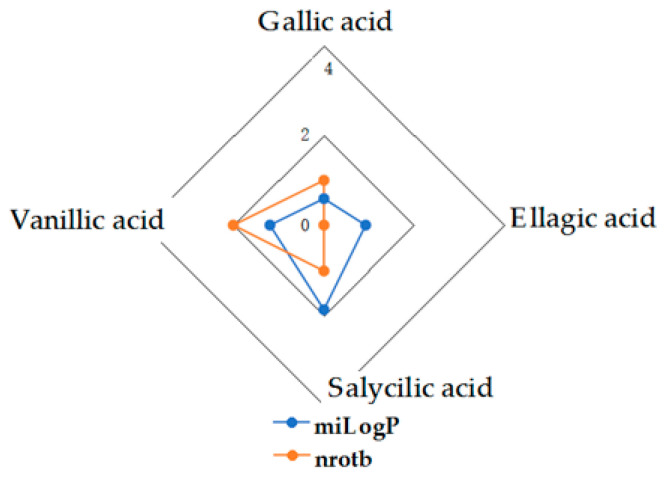
MiLog*P* along with nrotb for HBAc derivatives.

Applied to the present study, the computational analysis on milog*P*, along with nrotb (Figure 1, Figure 2, Figure 3, Figure 4, Figure 5, Figure 6, Figure 7, Figure 8 and Figure 9), suggested the following correlations between the bioavailability of the fifty compounds in the series: flavonols and flavones subclasses’ bioavailability generally increased with a decreasing number of hydroxyl groups at C and B rings (the summed milog*P* values of similar derivatives in each series decreased from the myricetin to kaempferol series and from luteolin to apigenin series, explained by the decrease in the number of hydroxyl groups at flavan core); flavan, flavanone and isoflavone derivatives generally had a better bioavailability than flavone and flavonol derivatives; in the flavonol series, the best bioavailability values were shown by aglycones followed by the -3-*O*-rhamnoside, -3-*O*-galactoside, -3-*O*-glucoside and -3-*O*-rutinoside series; in the flavone series, the best bioavailability was revealed by aglycones followed by -6/8-*C*-glucoside, -7-*O*-glucoside, -5-*O*-glucoside and -7-*O*-diglycoside; rutinoside and, generally, diglycoside derivatives revealed a lower bioavailability than monoglycoside derivatives, while rhamnoside derivatives largely showed the best bioavailability among the monoglycosides studied; genistein derivatives showed the best bioavailability values among the isoflavone series tested. In the phenylcarboxylic acid series, good bioavailability results were noticed in the hydroxybenzoic acid series; in the hydroxycinnamic acid series, aglycone compounds, especially curcumin, but also rosmarinic acid in comparison with other esterified homologues, appeared to have the best ability to pass the cell membranes in humans.

For further comparison with in vitro and in vivo data, it should be first noted that previous pharmacological studies demonstrated that the bioavailability of the plant compounds administered orally is the result of numerous biological processes; for example, the bio-accessibility of the active compounds, their intestinal and hepatic metabolism in correlation with their transformation by gut microflora, the nature of conjugates after hepatic metabolization and their plasma kinetics and binding to albumin, as well as their absorption at the level of the target cell, their accumulation in the specific tissues and urinary and biliary excretion.

In this way, a comparison with bioavailability data resulting from computational in silico studies could be a useful tool for better understanding plant compounds and herbal-derived medicines but also food products in relation to their health benefits and limits [56,57,58]. Previous clinical data revealed a low bioavailability for most of the plant compounds investigated; it was stated that about 5 percent of the daily oral intake of polyphenols is absorbed and metabolized at the level of the intestine and liver. For the most part, vegetal polyphenols in glycoside and esterified forms are generally thought to be degraded by the intestinal microflora and largely excreted as feces, excepting small quantities which are absorbed and metabolized at the level of intestine and liver [59]; as general rule, polyphenol aglycones proved to have a better bioavailability at the level of digestive system in humans (due to their lipophilic character), but they are present only in very small quantities in natural vegetal sources. Therefore, the glycoside forms and esterified derivatives are the predominant polyphenol compounds in the human diet.

In the specific case of flavonoids, the type and the number of units in glycoside moiety both play a crucial role in a polyphenol compound’s bioavailability; for example, studies revealed that, while glucoside derivatives are largely absorbed and metabolized at the level of small intestine, galactosides, rhamnosides, arabinosides, xylosides and glucuronic acid derivatives, such as polyglycoside derivatives (e.g., rutinoside), are metabolized at the level of the colon. The process is assisted by the bacterial hydrolases from the microbiota: the only ones that can cut esters and release aglycones from their glycoside moieties [58]. Another parameter that influences the bioavailability of the plant compounds is their affinity for human serum albumin [58,59]. Studies also showed that, even if the lipophilic compounds pass more successfully through the cell membranes, high hydrophilicity of a molecule increases the probability of binding to albumin; quercetin aglycone indicated the best interaction with albumin, explained by its planar conformation [56]. Concerning phenylcarboxylic acid derivatives’ bioavailability in vivo, data indicated that hydroxybenzoic acid derivatives are generally of low nutritional interest and, therefore, less studied; gallic acid is the most studied compound in the series, and it was proved to have high bioavailability in humans [58,59]. Hydroxycinnamic acid derivatives are of more nutritional interest, especially due to their transformation (e.g., hydrolysis to aglycones and multiple other isomerization transformations) during the sterilization, fermentation or freezing processes of fruits and vegetables. Studies revealed that, while the free aglycones are rapidly absorbed from the small intestine [60,61], the esterified forms (e.g., chlorogenic acid and its isomers) have a much lower bioavailability in humans [62,63,64], in the most part being metabolized by the intestinal hydrolases from microflora [65]. Finally, the high bioavailability noticed in vivo in the particular case of quercetin-4′-*O*-glucoside/spireoside from onions, of quercetin-3-*O*-glucoside/isoquercitrin from apples and of quercetin-3-*O*-galactoside/hyperoside from the St. John’s Wort herb was not sustained by the in silico computational study; these exceptions are explained by a cumulus of metabolic particularities and solvent effects, including the existence of specific intestinal hydrolases and the copresence of alcohol [58,66] or of pectins, surfactants and bitter compounds [67]. Similarly assigned as having good bioavailability in in silico studies, naringenin flavanone (found in citrus) can specifically increase the bioavailability of numerous xenobiotics in vivo by modulating the cytochrome P450 enzyme family function [68,69].

In summary, in silico results confirmed in vitro and in vivo data regarding the high bioavailability of soy isoflavones and better bioavailability of free aglycones in comparison with esterified and glycosylated forms. The computational study also revealed a high bioavailability for flavanones naringenin and hesperitin, apigenin and kaempferol derivatives and catechin and epicatechin flavan derivatives; curcumin, rosmarinic acid and salicylic acid were also revealed to have high bioavailability in the computational approach. In comparison, in vivo studies indicated the high bioavailability of caffeic and ferulic acids, while proanthocyanidols and gallocatechins (from green tea) were ranked last [58]; however, some clinical data proved anthocyanins (flavan derivatives) are fully absorped in humans [70]. Rhamnoside derivatives, the predominant polyphenolic compounds found in plant-derived products and the human diet [71], which were reported to have the lowest bioavailability by in vivo studies [72], were revealed to have the higher bioavailability values (miLog*P*/nrotb comparison) among the flavonoid series in the computational approach.

#### 2.1.1. Predicted Bioactivity

Table 10 gives the bioactivity scores predicted with Molinspiration software (Slovensky Grob, Slovak Republic: https://www.molinspiration.com (accessed on 2 June 2021) of the fifty test compounds towards six cell modulators, namely, G-protein-coupled receptors (GPCRs), ion channel modulators, kinase inhibitor, nuclear receptor, protease inhibitor and enzyme inhibitor activity. A high bioactivity score suggests a greater probability of a test molecule being active against a selected target. According to similar predictive studies [73,74], active molecules exhibit a bioactivity score of more than 0, moderately active between −5.0 and 0.0 and inactive less than −5.0. The general behavior and the compounds in the series with the highest activity (stimulatory activity) were, therefore, analyzed as follows.

Overall, comparative analysis of the fifty test compounds on the six cell modulators indicated good to moderate bioactivity scores; a remarkable similitude between the flavonoid series was also observed (see the supplementary material plotting their comparative activity areas, Figures S10–S15), the differences between the compounds in the series mostly being the intensity of the effects on the cell modulator.

Since G-protein-coupled receptors (GPCRs) belong to a large family of signaling proteins which mediate the cellular responses to numerous external molecules (ligands), such as hormones, cytokines, neurotransmitters and various metabolites, 34% of FDA-approved drugs target the 108 members of this family of cell surface receptors [75]. According to the literature data [76,77,78], GPCRs are involved in numerous physiological processes, including reactions upon the visual, gustatory and smell senses, behavioral and mood regulation, immune system regulation, autonomic nervous system transmission (responsible for the control of blood pressure, heart rate and digestive processes), cell density sensing, homeostasis modulation, tumor cell growth and metastasis and also hormone binding (through cAMP–kinase stimulation), thus, allowing the transcription processes in cells. The computational analysis carried out on the fifty polyphenolic compounds generally indicated moderate activity for flavonoid subclasses, the most active compounds against GPCR function being flavan derivatives, catechin and epicatechin, respectively (+0.41). Phenylcarboxylic acid derivatives were less active than flavonoid derivatives, apart from caffeic acid derivatives (chlorogenic and rosmarinic acid esters), which were shown to have higher potency (+0.29); compounds which less significantly affected the GPCRs’ activity were salicylic acid followed by vanillic acid > gallic acid > p-coumaric acid > caffeic acid > ferulic acid > sinapic acid > ellagic acid > quinic acid.

Ion channels are pore-forming membrane proteins which allow ions (e.g., calcium, potassium, sodium, chlorine) to pass through the channel pore, thus, principally controlling the flow of the ions across the cell membrane and, therefore, the electrolyte balance of the body and the cell volume. However, they are also involved in body cell signaling activity through ligand-gated ion channel signalization molecules, 5-HT3 (5-hydroxytriptamine receptor mediates neuronal depolarization and excitation), GABBA (gamma amino butyric acid is the major inhibitory neurotransmitter in the brain), glutamate (the most important neurotransmitter in the nervous system) and nicotinic receptor (helping the transmission of outgoing signals from the sympathetic and parasympathetic system to the whole body) [79,80,81]. The computational analysis indicated almost identical behavior in the case of GPCRs’ function: a generalized, moderate activity of the compounds tested and the same amplified activity against ion channel activity of flavan derivatives and caffeic acid derivatives. The less active compounds against ion channel activity were flavonoid rutinosides, isoflavones, flavanones and phenylcarboxylic acid aglycones. It must be noted that ion channel function controls every aspect of the digestion process (e.g., fluid secretion and absorption, motility and visceral sensitivity), irritable bowel syndrome manifestations being mostly driven by the altered ion channel expression and function [82]. These data are helpful in selecting the most appropriate plant-derived drug therapy in the situation of a susceptible patient.

Kinases are some of the most important enzymes in developing human body physiology since they catalyze the transfer of a phosphate group through which the high energy of an ATP molecule is donated as a phosphate group to a substrate molecule; this process is critical in all aspects of cell metabolism in prokaryotic processes, for example, cell signaling, protein regulation and cellular transport, all secretory processes and many other biological processes [83]. Related to the fifty compounds studied, all flavonoid compounds, particularly the aglycone forms in the series, indicated inhibitory potency against the activity of kinases (e.g., quercetin 0.28, myricetin 0.28, luteolin 0.26 and kaempferol 0.21); flavanone and isoflavone derivatives, similar to phenylcarboxylic acid aglycones (e.g., salicylic acid, vanillic acid, p-coumaric acid and gallic, caffeic and quinic acids), exhibited the weakest ability to influence the activity of human kinases.

The nuclear receptor superfamily comprises transcriptional factors (proteins) involved in thyroid/steroid hormones sensing. They can directly interact with the DNA molecule (by binding condensed chromatin templates); therefore, they control the gene expression and corresponding cell (embryo and adult) development, homeostasis and metabolism [84,85]. Their natural ligands are in the series of lipophilic substances (e.g., vitamins A and D) [86]; in the series of phytocompounds studied, the most augmented activity against nuclear receptor function (measuring from 0.57 to 0.74) was registered in the case of flavan, catechin and epicatechin and caffeic acid derivatives (e.g., chlorogenic acid, isochlorogenic acid, neochlorogenic acid > catechin, epicatechin > rosmarinic acid). Quercetin (0.36), hesperitin (0.38) and luteolin (0.39) also showed real inhibitory potency; the compounds with the weakest activity against nuclear receptor activity were in the series of hydroxybenzoic acid derivatives (e.g., salicylic acid, vanillic acid and gallic acid).

Protease inhibitors are basically compounds that can bind proteolytic enzymes (namely proteases) and block their function in the body. Digestion and healing wounds are two major examples of biological process which cannot be achieved without the activity of proteases, and HIV inhibitors are an example of potential use and applicability [87]. Related to the digestion process, studies indicated that patients taking protease inhibitors started to manifest important side effects such as “⋯new or exacerbated cases of diabetes or hyperglycemia, hemolytic anemia, spontaneous bleeding in hemophiliac patients, and changes in body composition.” [88]. All plant compounds tested indicated a moderate protease inhibitor activity (bioactivity scores less than 0), apigenin derivatives (from 0.01 to 0.04), catechins (+0.26) and caffeic acid derivatives (from 0.15 to 0.27) proving the highest scores.

Enzyme inhibitor activity is likely the parameter with the highest negative potential upon the digestion process, aside from ion channel inhibition. As shown in Table 10, all the polyphenolic compounds studied acted as enzyme inhibitors, the usually naturally occurring glycosylated and esterified forms being occasionally more active than aglycones forms. Computed as having a high bioactivity score in the investigated flavonoid series (0.43), an additional concern comes from quercetin-4′-*O*-glucoside (spireoside from onion), which is known to have the highest bioavailability in humans (counted at about 42% from the ingested weight). Aside from this, the most frequent polyphenol compounds in the foods to show the biggest bioactivity scores (0.62 and 0.47) were chlorogenic acid esters and flavan derivatives, together confirming the antinutritional potential of polyphenols in humans.

In support of these findings, studies on 21 flavonoid compounds indicated that luteolin, luteolin-7-*O*-glucoside, amentoflavone and daidzein were the most powerful alpha-glucosidase and alpha-amylase inhibitors, even stronger than acarbose [89]. Other studies on 14 compounds of plant origin indicated six phenolics with certain inhibitory activity upon trypsin activity, active at concentration values (IC_50_) ranging from 3.7 to 15.4 µM; they were silybin (3.7 µM), hypericin (4.5 µM), sennoside A and B (6.1 and 10.6 µM), hyperoside (14.5 µM) and quercetin (15.4 µM). Studies also demonstrated that a glycoside chain in position 3 of the flavan core led to high inhibitory potency [90]. Furthermore, a computer-assisted drug design (CADD) study upon 16 flavonoid compounds revealed that 5,7-dihydroxy derivatives were the best trypsin and trypsin-like enzyme inhibitors, quercetin, myricetin and morin compounds having the best structural configuration due to their suitably located hydroxyl groups and planar configuration as well. The specific compounds and their IC_50_ (µM) values were as follows: quercetin (10 µM), myricetin (15 µM), morin (27 µM), galangin (36 µM), isorhamnetin (40 µM), fisetin (46 µM), kaempferol (60 µM), acacetin (28 µM), apigenin (40 µM), baicalein (55 µM), 7,8-dihydroxyflavone (657 µM), chrysin (>1000 µM), 6/7-di-hydroxyflavones (>1000 µM), naringenin (484 µM) and biochanin A (134 µM) [47]. It was observed that quercetin had the lowest IC_50_ value in the series, meaning it had inhibitory potency upon the digestive protease enzymes at doses similar or lower than that of most beneficial activities in vitro.

Proving this, in vitro studies aiming to assess the defending activity of quercetin against cell death (endothelial cells, human skin fibroblasts and keratinocytes) induced by intracellular peroxides generated by buthionine sulfoximine (an irreversible inhibitor of glutathione synthesis) indicated that the protective effect of quercetin is manifested at EC_50_ values between 30 and 40 µM [91]. Studies regarding the antiarthritic, anti-inflammatory and antioxidant activity of nine South African plants used traditionally to treat arthritis [92], also revealed IC_50_ values from 11.89 to 53.78 µg/mL. Other studies [93] regarding the in vivo results (animal models) of the antidiabetic, anti-inflammatory, antioxidant, antimicrobial, anti-Alzheimer’s, antiarthritic, cardiovascular and wound-healing effects of quercetin administered orally in rats and mice indicated the following data: antidiabetic activity occurs at 10–100 mg/kg body, anti-Alzheimer activity at 10–50 mg/kg body, antiarthritic activity at 30 mg/kg body, antimicrobial effects at 5–30 mg/kg body, liver protection at 100 mg/kg body, antioxidant effects at 30 mg/kg body and protective cardiovascular effects up to 1.5 g/kg body; at the same time, in vitro results on different types of cancer cell indicated an inhibitory activity in the interval 5–50 µM. In summary, since the inhibitory activity of quercetin on the digestive enzymes (computed at IC_50_ = 10 µM) occurs at lower concentrations than that necessary for any other beneficial effect (most of values being over 10 µM in vitro and 10 mg/kg body in vivo), the food products and, especially, plant-based drugs based on quercetin and its derivatives could have this potential negative side effect on the digestion at humans.

Using the specialized database [94], Table S1 summarizes the mean content (mg per 100 g/mL) of the studied compounds in food products (e.g., cereals, fruits, vegetables, spices and herbs), highlighting the richest vegetal sources reported. The achieved analysis indicated that the sub(sub)classes of plant polyphenols in the present study mostly fell in the range of 0–50 mg per 100 g of product. The vegetal sources over 50 mg per 100 g of product were in the interest area for inhibitory potency, but the conclusion could be drawn by also considering the frequency and the amount of the product used in the daily diet of humans. In this way, in the series of flavonoid derivatives, flavan-3-ols were emphasized as the dominant polyphenolic compounds in the food products, while caffeic acid derivatives were likely the dominant bioactive compounds in the series of phenolic acid derivatives. It can be concluded that, excepting cocoa, chocolate, chestnut, coffee, tea (black, green), plum and berry products, which can each bring between 250 and 500 mg of a polyphenol subclass per day, all other food products do not reach high concentrations of specific polyphenol compounds in daily food. Thus, the main concern regarding plant polyphenol inhibitory activity on the digestive enzymes in humans is basically through the consumption of commercial products usually recommended in doses up to 1000 mg per day. In conclusion, the recommendation to supplement the usual diet with plant-derived products should be strictly made by specialists.

Finally, it is remarkable that birds developed several defense mechanisms to counteract plant polyphenols’ harmful, antinutritional effects, specifically by developing an alkaline pH gut, by secreting high contents of surfactants to decrease the polyphenols’ affinity in the intestine and by the presence of a peritrophic membranes and mucus able to absorb tannins after that excreted in the feces [95].

#### 2.1.2. Principal Component Analysis of the Test Parameter

Principal component analysis (PCA) is a statistical tool for the identification of linear combinations of the variables which account for certain proportions of the variance of the set of variables. The selection is based on the eigenvalues of the dispersion matrix of variables. The principal components are associated with decreasing eigenvalues and, therefore, share the amount of variance. Usually, the first few principal components account for virtually all the variances. PCA also represents the pattern of similarity of the observations and the variables by displaying them as points in maps [96,97,98,99]. The predicted bioactivity score data in Table 10 were processed by the PCA XLSTAT extension of Excel. The PCA correlation matrix (Table 11) showed a good correlation (r = 0.946) between GPCR and protease inhibitor parameter and a moderate correlation between GPCR and nuclear receptor (r = 0.799), enzyme inhibitor and nuclear receptor (r = 0.797) and enzyme inhibitor and protease inhibitor (r = 0.835), respectively.

**Figure 10:**
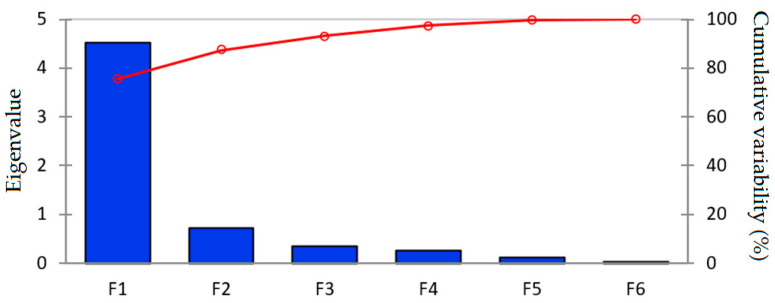
Scree plot of the eigenvalues and cumulative variability vs. the F1–F6 components.

Figure 10 and Table 12 are related to the eigenvalues which reflect the quality of the projection from the F-dimensional initial (F = 6) to a lower number of dimensions. From Table 12 it can be observed that the first eigenvalue equaled 4.528, representing 75.464% of the total variability. Each eigenvalue corresponds to a factor and each factor to one dimension. A factor is a linear combination of the initial variables, and all the factors are uncorrelated (r = 0). The eigenvalues and the corresponding factors are sorted by descending order of how much of the initial variability they represent (converted to %). Specifically, the first two factors allowed 87.33% of the initial variability of the data.

**Figure 11:**
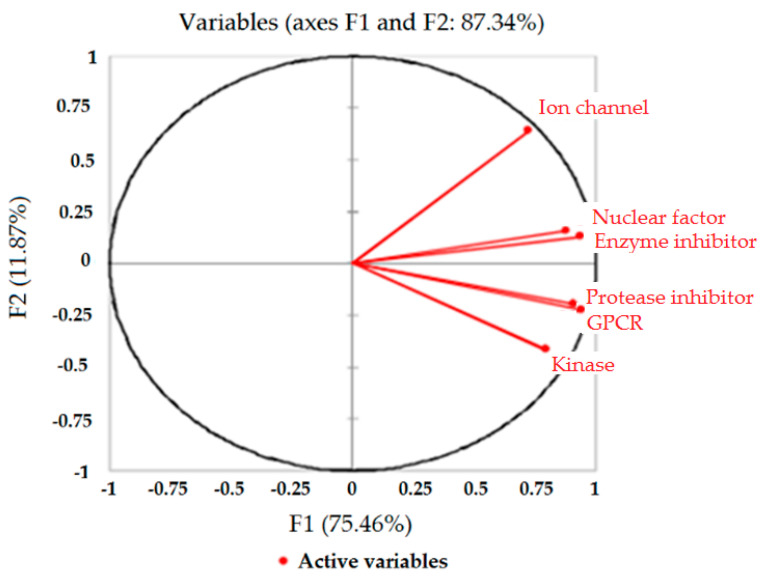
The correlation circle of F1 and F2 with the variables.

The correlation circle (Figure 11) below, on the axes F1 and F2, shows a projection of the initial variables in the factors space. When two variables are far from the center (as depicted for GPCR and nuclear receptor and enzyme inhibitor and nuclear receptor), if they are close to each other, they are significantly positively correlated (r close to 1); conversely, as observed for kinase and ionic channel, when they are almost orthogonal to each other, they are not correlated (r close to 0).

The correlation circle is also useful in interpreting the meaning of the axes. In this case, the horizontal axis is linked with GPCR, protease and enzyme inhibitor; as proof, in the squared cosines of the variables F1–F5 (Table 13), the greater the squared cosine, the greater the link with the horizontal axis.

## 3. Materials and Methods—Computational Procedure

### 3.1. Energy and Property Calculations

The investigated structures were firstly generated in 3D by importing their corresponding files from Pubchem database (https://pubchem.ncbi.nlm.nih.gov/ (accessed on 10 May 2020) in the Spartan’18 program [41,42]. Their geometry was optimized in a multi-step procedure by molecular mechanics force fields (MMFF, developed at Merck Pharmaceuticals) to obtain the lowest energy conformer corresponding to the most stable configuration of each structure [43]. Molecular properties and QSAR properties were calculated using density functional method, ωB97X-D (a range-separated hybrid generalized gradient approximation (RSH-GGA) functional) [44] with 6–31 G* polarization basis set. Computations were carried out for equilibrium geometry at ground state in gas of neutral molecules, without solvent corrections.

### 3.2. Property Calculations and Bioactivity Prediction Using Molinspiration Online Platform

In silico screening was realized using Molinspiration miscreen engine (Slovensky Grob, Slovak: https://www.molinspiration.com (accessed on 2 June 2021). Simplified molecular input line-entry system (SMILES) from Pubchem was used as input for property calculation engine. Bioactivity scores towards several active targets, protein-coupled receptor (GPCR) ligand, ion channel modulators, kinase inhibitors, nuclear receptor ligands, protease inhibitors and other enzyme targets, were predicted. Drug-likeness related parameters (the water–octanol partition coefficient (log*P*) and topological surface area (TPSA)) were also calculated.

### 3.3. Principal Component Analysis

The principal component analysis computations [96,97,98,99] were performed by a powerful, flexible Excel data analysis add-on provided by XLSTAT statistical software—Addinsoft (2020), XLSTAT statistical and data analysis solution, New York, NY, USA (https://www.xlstat.com (accessed on 6 August 2020).

## 4. Conclusions

Overall, in silico results confirmed in vitro and in vivo data regarding the high bioavailability of soy isoflavones and a better bioavailability of polyphenol aglycones in comparison with their esterified and glycosylated forms; specifically, the analysis of RO5 in relation to log*P* suggested that apigenin and apigenin-7-*O*-rhamnoside, naringenin, hesperetin, genistein, daidzin, biochanin A and formonetin in the flavonoid series and all hydroxycinnamic acids and all hydroxybenzoic acids excepting the condensed form of ferulic acid (namely ellagic acid) had the best bioavailability proofs in the computational approach. Rhamnoside monoglycosides were revealed to have the higher bioavailability values among the studied flavonoid series. In addition, considering the major contribution in relation to the oral bioavailability of an exogenous compound, the results of DFT computations indicated that the smallest deviations from the spherical shape were shown by quercetin, kaempferol, myricetin, luteolin, apigenin, naringenin, biochanin A, quinic acid, p-coumaric acid and salicylic acid; these data can be seen in the completion of another CADD study upon 16 flavonoids compounds which concluded that quercetin, myricetin and morin were the most active based on their suitably located hydroxyl groups and planar configuration too. Furthermore, the flexibility of a molecule (nrotb parameter) plays an important role in establishing interactions within the amino acids from the active binding site of an enzyme; no rotatable bond on the structure indicates rigid molecules. Relative to the fifty test compounds, the computational analysis indicated that the more flexible compounds were those containing rutinoside, glucoside and galactoside residues, followed by rhamnoside residue; curcumin, rosmarinic acid, chlorogenic acid and isochlorogenic acids, aside from sinapic acid, were also flexible molecules. On the other hand, a planar conformation of a molecule resulted in a better interaction with serum albumin and, therefore, in a better bioavailability in humans. In this way, the bioavailability of a vegetal compound is the result of an interplay of numerous physical, chemical, biological and microbiota characteristics in human.

The computational study on the investigated six parameters (GPCR, ion channel, kinase, nuclear receptor, protease inhibitor and enzyme cell modulators) indicated, overall, a remarkable similitude between the flavonoid series, flavonoid derivatives being more powerful natural cell modulators than the tested phenylcarboxylic acids. Specifically, the most active compounds against GPCR function were flavan derivatives; phenylcarboxylic acid derivatives were less active than flavonoid derivatives, apart from caffeic acid derivatives (chlorogenic acid, isochlorogenic acid, neochlorogenic acid and rosmarinic acid), which were shown to have a more augmented inhibitory potency. The analysis on the ion channel activity indicated the same amplified activity of flavan derivatives and caffeic acid derivatives; the less active compounds against ion channel activity were flavonoid rutinosides, isoflavones, flavanones and phenylcarboxylic acid aglycones. It must be noted that ion channel function regulates every aspect of the digestion process, irritable bowel syndrome manifestations being mostly driven by the altered ion channel expression and function. Studies regarding kinase activity indicated: polyphenol aglycones had higher inhibitory activity; the most potent compounds against ion channel activity were quercetin, myricetin, luteolin and kaempferol; and flavanone and isoflavone derivatives, such as phenylcarboxylic acid aglycones, demonstrated the weakest ability to influence the activity of human kinases. Nuclear receptor function analysis indicated flavan and caffeic acid derivatives had higher inhibitory potency; the compounds with the weakest activity upon nuclear receptor were in the series of hydroxybenzoic acid derivatives. Furthermore, all tested compounds indicated moderate protease inhibitor activity, apigenin derivatives, flavan derivatives and caffeic acid derivatives proving the highest bioactivity scores. Additionally, all the investigated compounds demonstrated the ability to act as enzyme inhibitors in humans, the naturally occurring glycosylated and esterified forms being more active than less current aglycone forms; chlorogenic acid esters and flavan derivatives showed the biggest bioactivity scores, together confirming the potential side effects of polyphenol compounds in humans.

Refining bioactivity data and the PCA correlation matrix also proved a good correlation between GPCR and protease inhibitor capacity for the polyphenolic compounds and a moderate correlation between GPCR and nuclear receptor, enzyme inhibitor capacity and nuclear receptor effects and enzyme and protease inhibitor capacity, respectively.

Therefore, the need to supplement with digestive enzymes, especially in people with low digestive efficiency due to multiple causes, should be considered in order to obtain the best benefits of vegetal polyphenols for human health.

## Figures and Tables

**Table 1:** Predicted molecular properties for quercetin (Q) and quercetin derivatives (see Figure S1 in supplementary materials).

Property	Q	Q-3-*O*-Gal	Q-3-*O*-Glc	Q-3-*O*-Rhm	Q-3-*O*-Rut	Q-4′-*O*-Glc	Q-7-*O*-Glc
Molecular weight (Da)	302.238	464.379	464.379	448.380	610.521	464.379	464.379
Area (Å^2^)	278.15	413.87	402.49	406.58	536.48	420.58	422.15
Volume (Å^3^)	264.73	400.50	398.89	394.14	530.88	401.57	401.50
PSA (Å^2^)	108.359	171.154	165.115	157.308	227.089	175.445	178.130
TPSA (Å^2^) *	131.35	210.50	210.50	190.28	269.43	210.50	210.50
Ovality	1.40	1.58	1.54	1.56	1.69	1.60	1.60
Polarizability (10^−30^ m^3^)	61.02	72.02	71.93	71.50	82.59	72.13	72.12
Dipole moment (Debye)	7.09	9.91	4.69	6.73	7.21	5.83	5.35
log*P*	−4.54	−6.54	−6.54	−5.68	−7.42	−6.54	−6.54
miLog*P* *	1.68	−0.36	−0.36	0.64	−1.06	−0.33	−0.10
nrotb	1	4	4	3	6	4	4
RO5 violations	0	2	2	2	3	2	2

* TPSA and miLog*P* are predicted with Molinspiration.

**Table 2:** Predicted molecular properties for kaempferol (K) and kaempferol derivatives (see Figure S2).

Property	K	K-3-*O*-Gal	K-3-*O*-Glc	K-3-*O*-Rhm	K-3-*O*-Rut
Molecular weight (Da)	286.239	448.380	448.380	448.381	594.522
Area (Å^2^)	270.77	406.20	395.43	402.03	531.09
Volume (Å^3^)	258.01	393.79	392.24	388.62	524.07
PSA (Å^2^)	90.833	153.214	147.930	143.262	209.216
TPSA (Å^2^) *	111.12	190.28	190.28	170.05	249.20
Ovality	1.38	1.56	1.53	1.56	1.69
Polarizability (10^−30^ m^3^)	60.47	71.46	71.36	7.98	82.00
Dipole moment (Debye)	4.47	10.70	5.90	7.44	6.13
log*P*	−3.46	−5.45	−5.45	−4.60	−4.34
miLog*P* *	2.17	0.12	0.12	1.13	−0.57
nrotb	1	4	4	3	6
RO5 violations	0	2	2	1	3

* TPSA and miLog*P* are predicted with Molinspiration.

**Table 3:** Predicted molecular properties for myricetin (M) and myricetin derivatives (see Figure S3).

Property	M	M-3-*O*-Gal	M-3-*O*-Glc	M-3-*O*-Rhm	M-3-*O*-Rut
Molecular weight (Da)	318.237	480.378	480.378	464.379	626.52
Area (Å^2^)	286.39	414.73	413.97	413.29	527.79
Volume (Å^3^)	271.76	407.21	405.91	400.46	533.82
PSA (Å^2^)	127.301	188.639	181.392	171.358	221.943
TPSA (Å^2^) *	151.58	230.73	230.73	210.50	289.65
Ovality	1.41	1.56	1.56	1.57	1.66
Polarizability (10^−30^ m^3^)	61.59	72.61	72.60	72.01	82.83
Dipole moment (Debye)	6.66	1.87	4.85	8.46	3.00
log*P*	−5.63	−7.62	−7.62	−6.77	−8.51
miLog*P* *	1.39	−0.66	−0.66	0.35	−1.35
nrotb	1	4	4	3	6
RO5 violations	1	2	2	2	3

* TPSA and miLog*P* are predicted with Molinspiration.

**Table 4:** Predicted molecular properties for luteolin (L) and luteolin derivatives (see Figure S4).

Property	L	L-5-*O*-Glc	L-7-*O*-Glc	L-6-*C*-Glc	L-8-*C*-Glc	L-7-*O*-Rut	L-7,3′-Di-*O*-Glc
Molecular weight (Da)	286.239	448.38	448.38	448.38	448.38	594.522	610.521
Area (Å^2^)	271.48	417.67	415.37	393.20	392.15	538.70	535.04
Volume (Å^3^)	258.05	396.39	394.81	389.33	390.32	524.03	529.13
PSA (Å^2^)	90.988	162.897	160.669	154.336	160.037	208.614	209.915
TPSA (Å^2^) *	111.12	190.28	190.28	201.27	201.27	249.20	269.43
Ovality	1.38	1.60	1.60	1.52	1.52	1.71	1.69
Polarizability (10^−30^ m^3^)	60.44	71.63	71.54	71.11	71.20	81.99	52.44
Dipole moment (Debye)	4.47	4.99	6.85	11.48	6.82	7.63	9.85
log*P*	−3.46	−5.45	−5.45	−6.39	−6.39	−6.34	−2.67
miLog*P* *	1.97	−0.07	0.19	0.03	0.03	−0.51	−1.83
nrotb	1	4	4	3	3	6	7
RO5 violations	0	2	2	2	2	3	3

* TPSA and miLog*P* are predicted with Molinspiration.

**Table 5:** Predicted molecular properties for apigenin (A) and apigenin derivatives (see Figure S5).

Property	A	A-5-*O*-Glc	A-7-*O*-Glc	A-6-*C*-Glc	A-8-*C*-Glc	A-7-Rut	A-7-Rhm
Molecular weight (Da)	270.24	432.381	432.381	432.381	432.381	578.523	416.382
Area (Å^2^)	263.92	402.21	407.69	395.93	400.77	525.09	394.21
Volume (Å^3^)	251.24	387.61	397.98	384.11	385.88	514.70	380.12
PSA (Å^2^)	73.293	136.935	142.854	144.153	151.736	173.833	117.807
TPSA (Å^2^) *	90.89	170.05	170.05	181.04	181.04	228.97	149.82
Ovality	1.37	1.56	1.58	1.55	1.56	1.69	1.55
Polarizability (10^−30^ m^3^)	59.87	70.90	70.96	70.65	70.80	81.20	70.32
Dipole moment (Debye)	3.58	6.70	6.68	5.34	4.31	5.93	5.45
log*P*	−2.38	−4.37	−4.37	5.31	−5.31	−5.26	−3.52
miLog*P* *	2.46	0.42	0.68	0.52	0.52	−0.02	1.68
nrotb	1	4	4	3	3	6	3
RO5 violations	0	1	1	1	1	3	0

* TPSA and miLog*P* are predicted with Molinspiration.

**Table 6:** Predicted molecular properties for flavanone and flavan derivatives (see Figure S6).

Property	Naringenin (N)	*N*-7-Rutinoside	Hesperitin (H)	*H*-7-Rutinoside	Catechin	Epicatechin
Molecular weight (Da)	227.25	580.539	302.282	610.565	290.271	290.271
Area (Å^2^)	273.50	541.63	302.71	558.82	287.52	284.97
Volume (Å^3^)	257.30	520.93	284.46	545.73	268.97	268.38
PSA (Å^2^)	77.897	187.032	83.737	186.033	102.102	100.445
TPSA (Å^2^) *	86.99	225.06	96.22	234.30	110.37	110.37
Ovality	1.40	1.73	1.45	1.73	1.43	1.42
Polarizability (10^−30^ m^3^)	60.17	81.65	62.40	83.72	60.94	60.95
Dipole moment (Debye)	3.51	7.88	3.94	5.73	2.20	1.10
log*P*	−2.15	−5.03	−3.12	−6.00	−3.72	−3.72
miLog*P* *	2.12	−0.37	1.94	−0.55	1.37	1.37
nrotb	1	6	2	7	1	1
RO5 violations	0	3	0	3	0	0

* TPSA and miLog*P* are predicted with Molinspiration.

**Table 7:** Predicted molecular properties for isoflavone derivatives (see Figure S7).

Property	Genistein	Formonetin	Biochanin A	Daidzin
Molecular weight (Da)	270.24	268.268	284.267	416.382
Area (Å^2^)	262.40	278.51	283.07	401.92
Volume (Å^3^)	251.09	265.31	270.96	382.21
PSA (Å^2^)	73.021	40.36	60.276	128.677
TPSA (Å^2^) *	90.89	59.67	79.90	149.82
Ovality	1.36	1.39	1.40	1.58
Polarizability (10^−30^ m^3^)	59.85	61.01	61.47	70.48
Dipole moment (Debye)	1.39	1.75	1.32	5.15
log*P*	−2.03	−0.84	−1.92	−2.94
miLog*P* *	2.27	3.10	2.80	0.77
nrotb	1	2	2	4
RO5 violations	0	0	0	0

* TPSA and miLog*P* are predicted with Molinspiration.

**Table 8:** Predicted molecular properties for hydroxycinnamic acid (HCAc) derivatives (see Figure S8).

Property	Coumaric Acid	Ferulic Acid	Sinapic Acid	Curcumin	Caffeic Acid	Quinic Acid	Chlorogenic Acid	Neochlorogenic Acid	Isochlorogenic Acid	Rosmarinic Acid
Molecular weight (Da)	164.160	194.186	224.212	368.385	180.159	192.167	354.311	354.311	354.311	360.318
Area (Å^2^)	189.22	218.72	246.61	404.72	196.80	187.53	346.33	347.65	344.23	368.40
Volume (Å^3^)	166.22	193.46	220.08	375.31	173.06	167.16	320.18	320.70	320.45	337.46
PSA (Å^2^)	54.039	60.113	64.50	79.438	71.791	101.904	141.375	143.496	145.352	129.671
TPSA (Å^2^) *	57.53	66.76	76.00	93.07	77.75	118.21	164.74	164.74	164.74	144.52
Ovality	1.29	1.35	1.40	1.61	1.31	1.28	1.53	1.53	1.52	1.57
Polarizability (10^−30^ m^3^)	52.94	55.17	57.38	70.11	53.53	52.47	65.50	65.51	65.52	66.91
Dipole moment (Deb.)	3.38	3.30	3.21	3.65	4.79	3.47	5.10	3.47	5.84	3.19
log*P*	0.22	−0.75	−1.73	−0.46	−0.86	−2.09	−2.42	−2.42	−2.42	−2.13
miLog*P* *	1.43	1.25	1.26	2.30	0.94	−2.33	−0.45	−0.45	−0.45	1.63
nrotb	2	3	4	8	2	1	5	5	5	7
RO5 violations	0	0	0	0	0	0	1	1	1	0

* TPSA and miLog*P* are predicted with Molinspiration.

**Table 9:** Predicted molecular properties for hydroxybenzoic acids (HBAc) derivatives (see Figure S9).

Property	Gallic Acid	Salicylic Acid	Vanillic Acid	Ellagic Acid
Molecular weight (Da)	170.120	138.122	168.148	302.194
Area (Å^2^)	169.47	152.90	182.90	251.80
Volume (Å^3^)	147.39	133.62	160.77	243.28
PSA (Å^2^)	88.921	51.351	57.449	115.461
TPSA (Å^2^) *	97.98	57.53	66.76	141.33
Ovality	1.26	1.21	1.28	1.34
Polarizability (10^−30^·m^3^)	51.25	50.08	52.35	59.29
Dipole moment (Debye)	2.25	2.74	4.09	4.90
log*P*	−2.46	−0.29	−1.27	−5.33
miLog*P* *	0.59	1.87	1.19	0.94
nrotb	1	1	2	0
RO5 violations	0	0	0	0

* TPSA and miLog*P* are predicted with Molinspiration.

**Table 10:** Predicted bioactivity scores, using Molinspiration engine.

Plant Compounds	GPCR	Ion Channel	Kinase	Nuclear Receptor	Protease Inhibitor	Enzyme Inhibitor
Quercetin derivatives						
Quercetin	−0.06	−0.19	0.28	0.36	−0.25	0.28
Quercetin-3-*O*-galactoside	0.06	−0.04	0.13	0.20	−0.06	0.42
Quercetin-3-*O*-glucoside	0.06	−0.04	0.13	0.20	−0.06	0.42
Quercetin-3-*O*-rhamnoside	−0.01	−0.08	0.08	0.17	−0.06	0.37
Quercetin-3-*O*-rutinoside	−0.05	−0.52	−0.14	−0.23	−0.07	0.12
Quercetin-4′-*O*-glucoside	−0.05	−0.09	0.18	0.24	−0.07	0.43
Quercetin-7-*O*-glucoside	0.04	−0.10	0.15	0.23	−0.06	0.42
Kaempferol derivatives						
Kaempferol	−0.10	−0.21	0.21	0.32	−0.27	0.26
Kaempferol-3-*O*-galactoside	0.06	−0.05	0.10	0.20	−0.05	0.41
Kaempferol-3-*O*-glucoside	0.06	−0.05	0.10	0.20	−0.05	0.41
Kaempferol-3-*O*-rhamnoside	−0.01	−0.09	0.05	0.16	−0.05	0.36
Kaempferol-3-*O*-rutinoside	−0.01	−0.43	−0.09	−0.17	−0.04	0.18
Myricetin derivatives						
Myricetin	−0.06	−0.18	0.28	0.32	−0.20	0.30
Myricetin-3-*O*-galactoside	0.04	−0.04	0.13	0.17	−0.06	0.43
Myricetin-3-*O*-glucoside	0.04	−0.04	0.13	0.17	−0.06	0.43
Myricetin-3-*O*-rhamnoside	−0.02	−0.08	0.08	0.14	−0.06	0.38
Myricetin-3-*O*-rutinoside	−0.11	−0.62	−0.21	−0.34	−0.08	0.06
Luteolin derivatives						
Luteolin	−0.02	−0.07	0.26	0.39	−0.22	0.28
Luteolin-5-*O*-glucoside	0.12	0	0.18	0.29	−0.01	0.41
Luteolin-7-*O*-glucoside	0.09	−0.02	0.15	0.27	−0.01	0.42
Luteolin-6-*C*-glucoside	0.11	0.01	0.16	0.20	0.01	0.46
Luteolin-8-*C*-glucoside	0.12	−0.14	0.20	0.20	0.01	0.45
Luteolin-7-*O*-beta-rutinoside	0.01	−0.41	−0.05	−0.11	−0.01	0.18
Luteolin-7,3′-di-*O*-glucoside	−0.03	−0.50	−0.11	−0.06	−0.04	0.07
Apigenin derivatives						
Apigenin	−0.07	−0.09	0.18	0.34	−0.25	0.26
Apigenin-5-*O*-glucoside	0.11	−0.01	0.15	0.28	0.01	0.40
Apigenin-7-*O*-glucoside	0.10	−0.01	0.14	0.31	0.02	0.43
Apigenin-6-*C*-glucoside	0.12	0.02	0.15	0.23	0.04	0.47
Apigenin-8-*C*-glucoside	0.13	−0.14	0.19	0.23	0.03	0.46
Apigenin-7-rutinoside	0.05	−0.32	−0.01	−0.03	0.01	0.24
Apigenin 7-rhamnoside	0.03	−0.06	0.09	0.28	0.02	0.38
Flavanone derivatives						
Naringenin	0.03	−0.20	−0.26	0.42	−0.12	0.21
Naringenin-7-rutinoside	0.10	−0.37	−0.22	0	0.07	0.22
Hesperitin	0.04	−0.26	−0.20	0.38	−0.13	0.16
Hesperitin-7-rutinoside	−0.01	−0.59	−0.36	−0.20	0	0.06
Flavan derivatives						
Catechin	0.41	0.14	0.09	0.60	0.26	0.47
Epicatechin	0.41	0.14	0.09	0.60	0.26	0.47
Isoflavone derivatives						
Genistein	−0.22	−0.54	−0.06	0.23	−0.68	0.13
Daidzin	−0.01	−0.36	−0.07	0.14	−0.31	0.29
Biochanin A	−0.23	−0.59	−0.07	0.23	−0.66	0.07
Formonetin	−0.30	−0.69	−0.19	0.05	−0.8	−0.02
Hydroxycinnamic acid (C6-C3) derivatives						
p-coumaric acid	−0.56	−0.26	−0.91	−0.12	−0.87	−0.15
Ferulic acid	−0.47	−0.30	−0.72	−0.14	−0.81	−0.12
Curcumin	−0.06	−0.20	−0.26	0.12	−0.14	0.08
Caffeic acid	−0.48	−0.23	−0.81	−0.10	−0.79	−0.09
Quinic acid	−0.24	0.10	−0.77	0.16	−0.26	0.60
Chlorogenic acid	0.29	0.14	0	0.74	0.27	0.62
Neochlorogenic acid	0.29	0.14	0	0.74	0.27	0.62
Isochlorogenic acid	0.29	0.14	0	0.74	0.27	0.62
Rosmarinic acid	0.17	−0.08	−0.18	0.57	0.15	0.24
Sinapic acid	−0.32	−0.20	−0.47	−0.03	−0.56	0.03
Hydroxybenzoic acid (C6-C1) derivatives						
Gallic acid	−0.77	−0.26	−0.88	−0.52	−0.94	−0.17
Ellagic acid	−0.29	−0.27	−0.01	0.11	−0.18	0.17
Salicylic acid	−0.98	−0.43	−1.22	−0.79	−1.14	−0.41
Vanillic acid	−0.85	−0.42	−0.99	−0.61	−1.12	−0.35

**Table 11:** Correlation matrix of PCA *.

Variables	GPCR	Ion Channel	Kinase	Nuclear Receptor	Protease Inhibitor	Enzyme Inhibitor
GPCR	1.000	0.528	0.774	0.799	0.946	0.847
Ion channel	0.528	1.000	0.383	0.666	0.531	0.725
Kinase	0.774	0.383	1.000	0.629	0.683	0.654
Nuclear receptor	0.799	0.666	0.629	1.000	0.679	0.797
Protease inhibitor	0.946	0.531	0.683	0.679	1.000	0.835
Enzyme inhibitor	0.847	0.725	0.654	0.797	0.835	1.000

* r means the Pearson correlation coefficient.

**Table 12:** Eigenvalues from the PCA analysis.

	F1	F2	F3	F4	F5	F6
Eigenvalue	4.528	0.712	0.348	0.263	0.124	0.025
Variability (%)	75.464	11.872	5.806	4.381	2.059	0.419
Cumulative %	75.464	87.336	93.142	97.523	99.581	100.000

**Table 13:** Squared cosines of the variables *.

Variables	F1	F2	F3	F4	F5
GPCR	0.901	0.051	0.020	0.005	0.009
Ion channel	0.523	0.409	0.006	0.048	0.013
Kinase	0.628	0.173	0.157	0.041	0.000
Nuclear receptor	0.773	0.024	0.039	0.162	0.001
Protease inhibitor	0.824	0.038	0.114	0.005	0.010
Enzyme inhibitor	0.878	0.017	0.013	0.001	0.091

* values in bold correspond for each variable to the factor for which the squared cosine is the largest.

## Data Availability

Not applicable.

## Supplementary Materials

The following are available online, Figure S1: Optimized structures of quercetin (Q) and quercetin derivatives; Figure S2: Optimized structures of kaempferol (K) and kaempferol derivatives; Figure S3: Optimized structures of myricetin (M) and myricetin derivatives; Figure S4: Optimized structures of luteolin (L) and luteolin derivatives; Figure S5: Optimized structures of apigenin (A) and apigenin derivatives; Figure S6: Optimized structures of flavanone (naringenin/N and hesperitin/H) derivatives; Figure S7: Optimized structures of isoflavones derivatives; Figure S8: Optimized structures of hydroxycinnamic acid (HCAc) derivatives; Figure S9: Optimized structures of hydroxybenzoic acid (HBAc) derivatives; Figure S10: Plots of ion GPCD ligand scores; Figure S11: Plots of ion channel modulator scores; Figure S12: Plots of kinase inhibitor scores; Figure S13: Plots of nuclear receptor ligand scores; Figure S14: Plots of protease inhibitor scores; Figure S15: Plots of enzyme inhibitor scores; Table S1: The content of the fifty studied compounds in food products.

## Sample Availability

Samples of the compounds are not available from the authors.

## Abbreviations

A	Apigenin
CADD	Computer-assisted drug design
COX-1	Ciclooxigenase-1
COX-2	Ciclooxigenase-2
DFT	Density functional theory
E HOMO	Energy of the highest occupied molecular orbital
E LUMO	Energy of the lowest unoccupied molecular orbital
Egap	Energy gap between frontier molecular orbitals
FMOs	Frontier molecular orbitals
GPCRs	G-protein-coupled receptors
H	Hesperitin
HBA	Hydrogen bond acceptor
HBD	Hydrogen bond donor
HBAc	Hydroxybenzoic acids
HCAc	Hydroxycinnamic acids
HOMO	Highest occupied molecular orbital
K	Kaempferol
L	Luteolin
Log*P*	Octanol/water partition coefficient calculated with Spartan software
LUMO	Lowest unoccupied molecular orbital
M	Myricetin
MMFF	Molecular mechanics force fields
milogP	Octanol/water partition coefficient calculated with Molispiration
N	Naringenin
nrotb	Number of rotatable bonds
PCA	Principal component analysis
PSA	Polar surface area
r	Pearson correlation coefficient
RO5	Rule of five (Lipinski’s rule)
RSH-GGA	Range-separated hybrid generalized gradient approximation
TPSA	Topological polar surface area
Q	Quercetin
QSPR	Quantitative structure-property relationships
