# Supplementary material for: In Silico Study Approach on a Series of 50 Polyphenolic Compounds in Plants; A Comparison on the Bioavailability and Bioactivity Data
Source: Molecules. 2022 Feb 19;27(4):1413. doi: 10.3390/molecules27041413 (PMC8878759; doi:10.3390/molecules27041413)
Supplement: Supplementary file 1 [file molecules-27-01413-s001.zip › molecules-1540392-supplementary.pdf]

ELECTRONIC SUPPLEMENTARY MATERIAL

In silico study approach on a series of 50 polyphenolic compounds in plants; a comparison on the bioavailability and bioactivity data

Amalia Stefaniu[1*] and Lucia Camelia Pirvu[1*]

Correspondence to: astefaniu@gmail.com (A.S.); lucia.pirvu@yahoo.com (L.C.P.)

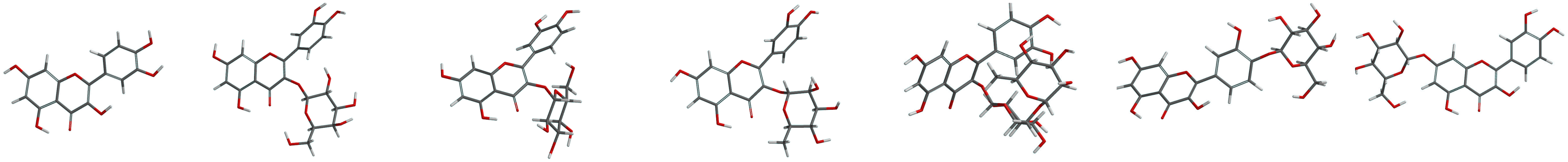

Quercetin **(Q)** **Q**-3-O-galactoside **Q**-3-O-glucoside **Q**-3-O-rhamnoside **Q**-3-O-rutinoside **Q**-4'-O-glucoside **Q**-7-O-glucoside

Figure S1. Optimized structures of Quercetin (**Q**) and Quercetin derivatives.

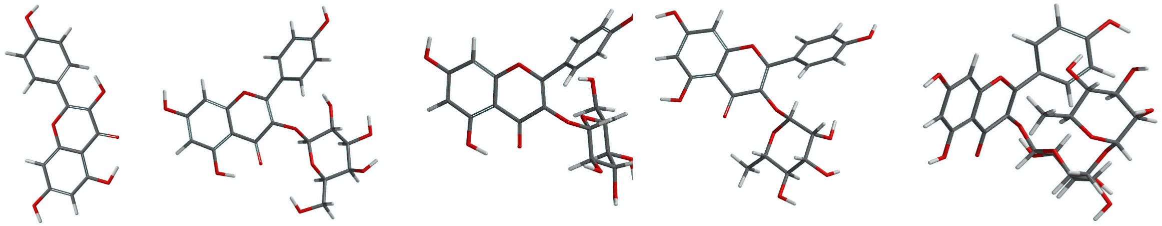

Kaempferol (**K**) **K**-3-O-galactoside **K**-3-O- glucoside **K**-3-O-rhamnoside **K**-3-O-rutinoside

Figure S2. Optimized structures of Kaempferol (**K**) and Kaempferol derivatives.

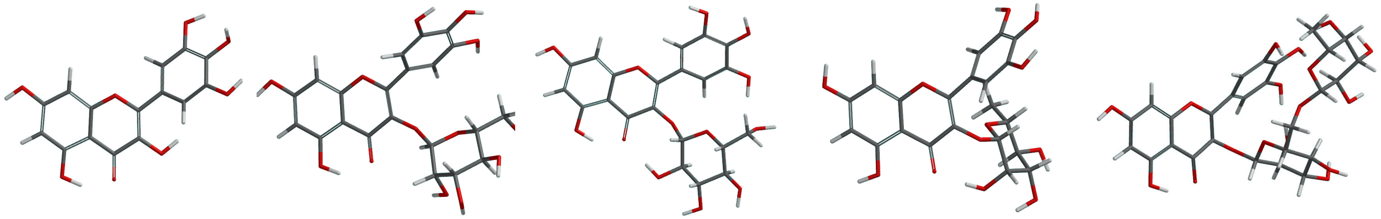

Myricetin **(M)** **M**-3-O-galactoside **M**-3-O-glucoside **M**-3-O-rhamnoside **M**-3-O-rutinoside

Figure S3. Optimized structures of Myricetin (**M**) and Myricetin derivatives.

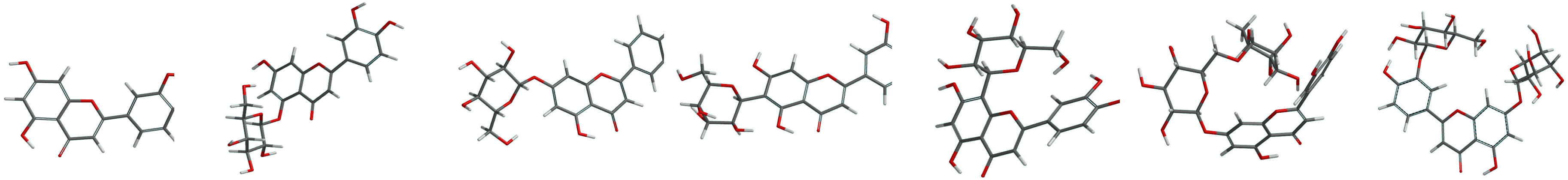

Luteolin **(L)** **L**-5-O-glucoside **L**-7-O-glucoside **L**-6-C-glucoside **L**-8-C-glucoside **L**-7-O-rutinoside **L**-7,3′-di-O-glucoside

Figure S4. Optimized structures of Luteolin (**L**) and Luteolin derivatives.

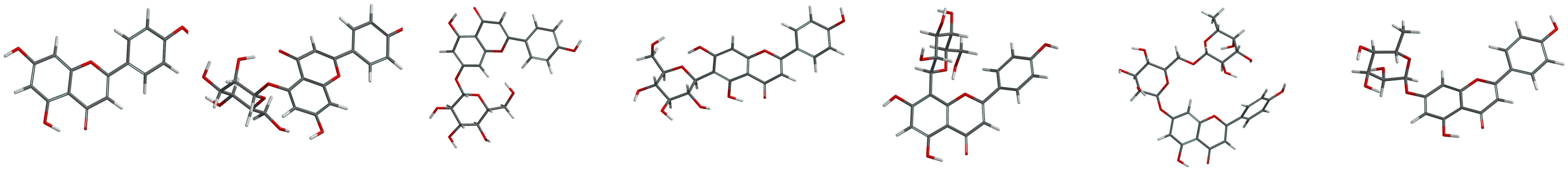

Apigenin **(A)** **A**-5-O-glucoside **A**-7-O-glucoside **A**-6-C-glucoside **A**-8-C-glucoside **A**-7- rutinoside **A**-7- rhamnoside

Figure S5. Optimized structures of Apigenin (**A**) and Apigenin derivatives.

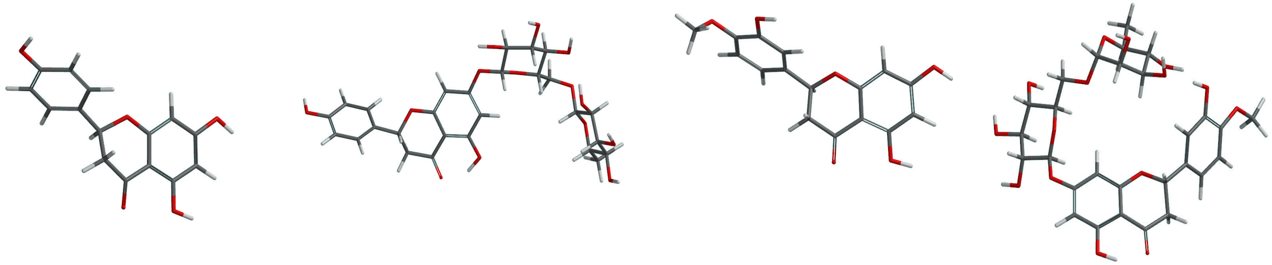

Naringenin (**N**) | **N**-7-rutinoside | Hesperitin (**H**) | **H**-7-rutinoside

Figure S6. Optimized structures of Flavanone (Naringenin/N and Hesperitin/H) derivatives.

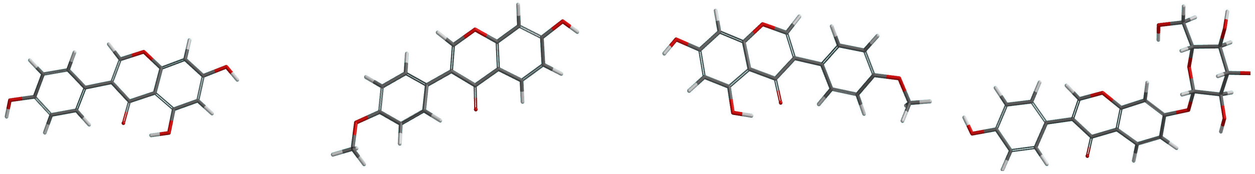

Genistein | Formonetin | Biochanin A | Daidzin

Figure S7. Optimized structures of Isoflavone derivatives.

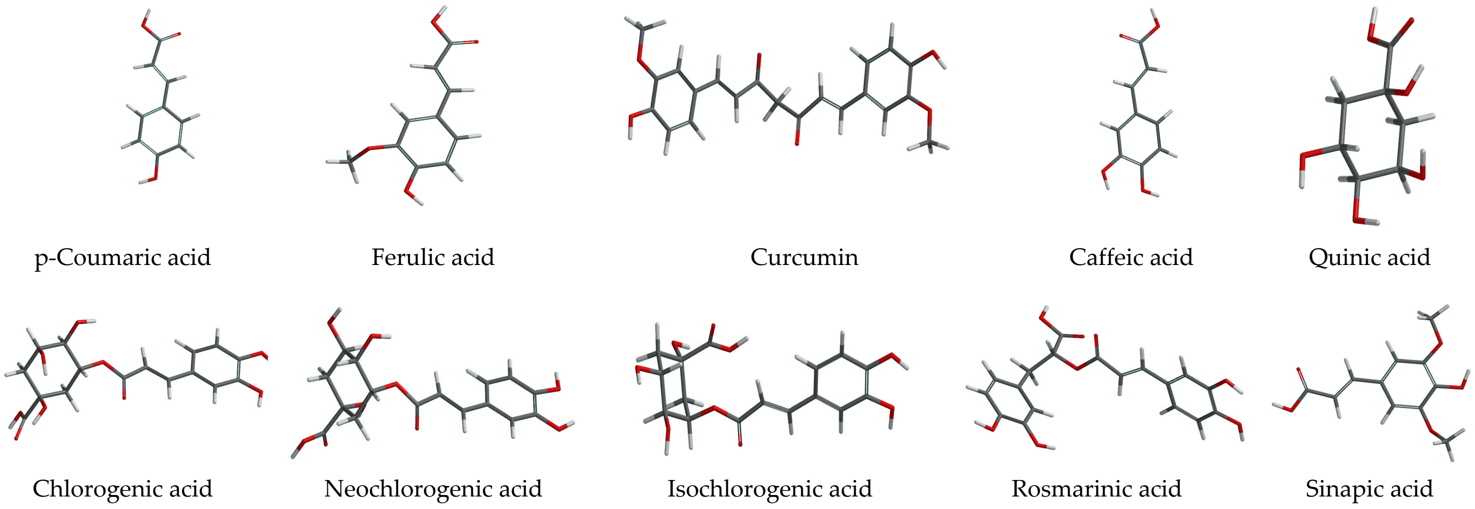

Figure S8. Optimized structures of Hydroxycinnamic acid (HCAc) derivatives.

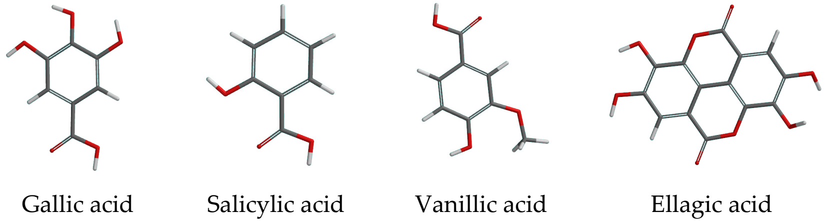

Figure S9. Optimized structures of Hydroxybenzoic acids (HBAc) derivatives.

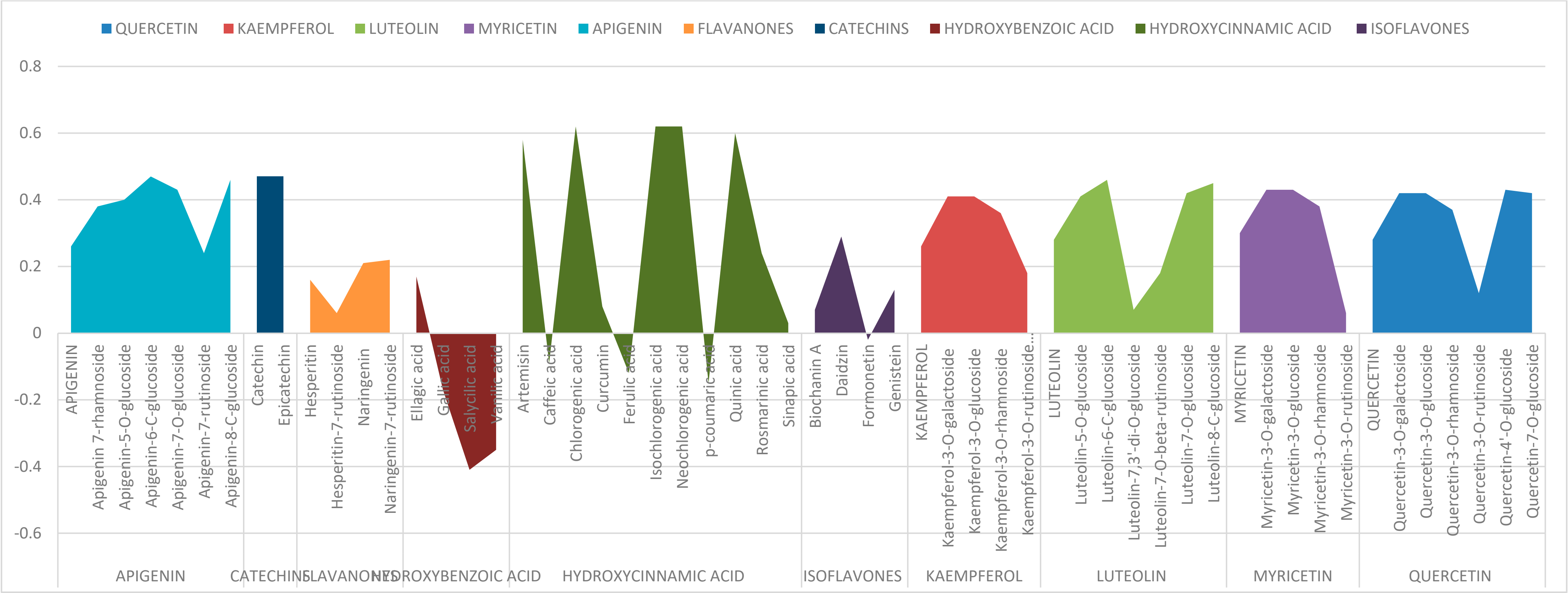

Figure S10. Plots of Ion GPCD ligand scores.

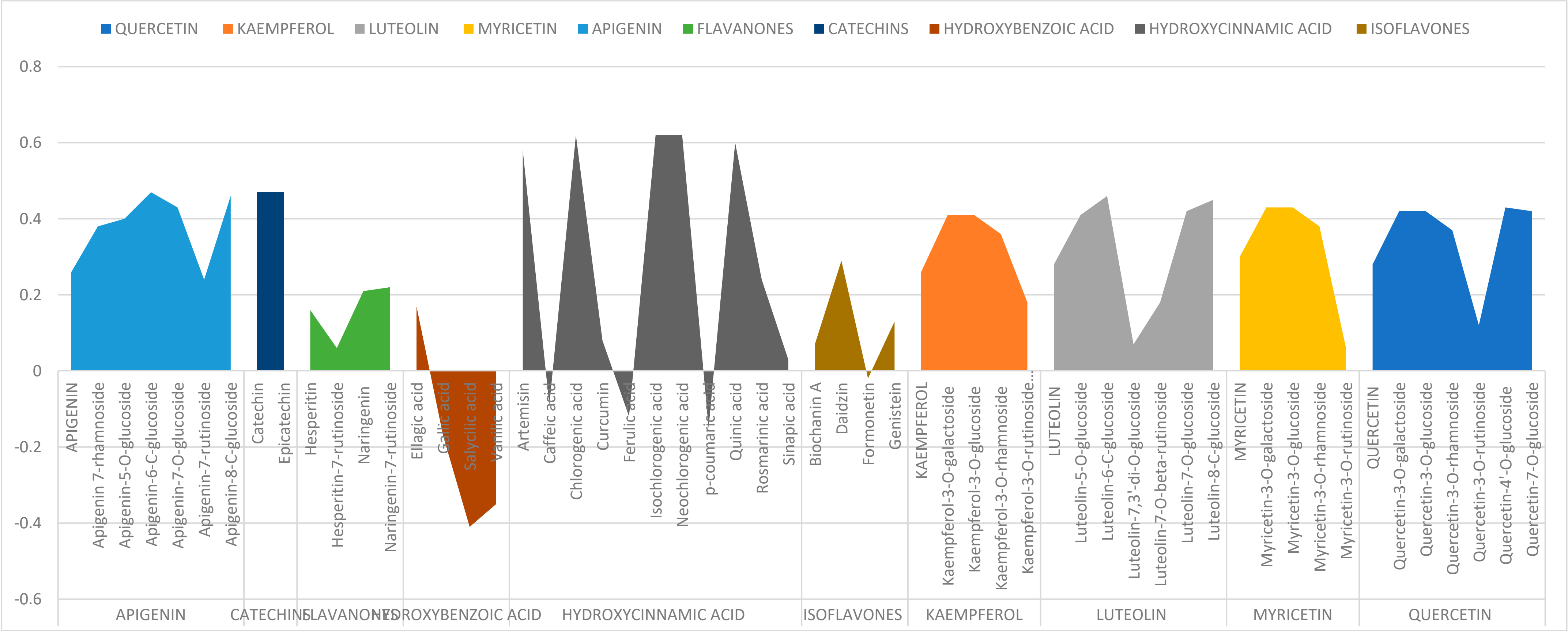

Figure S11. Plots of Ion Channel Modulator scores.

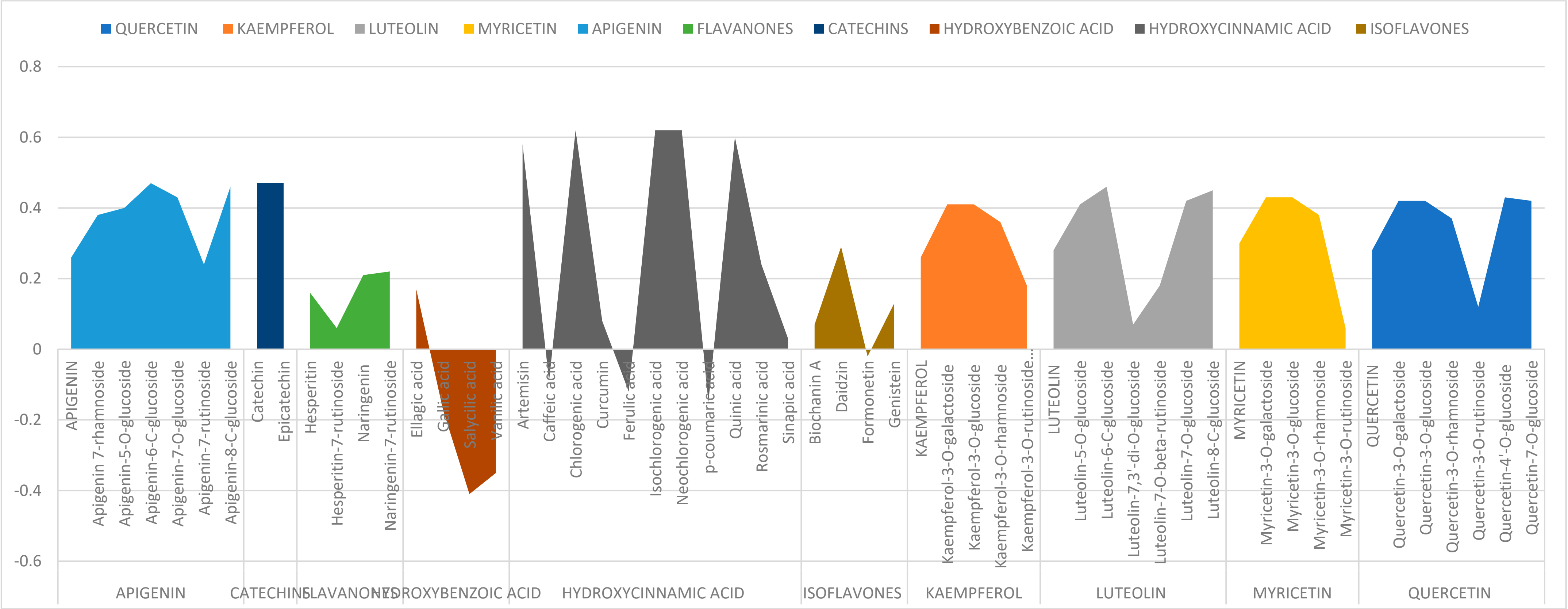

Figure S12. Plots of Kinase inhibitor scores.

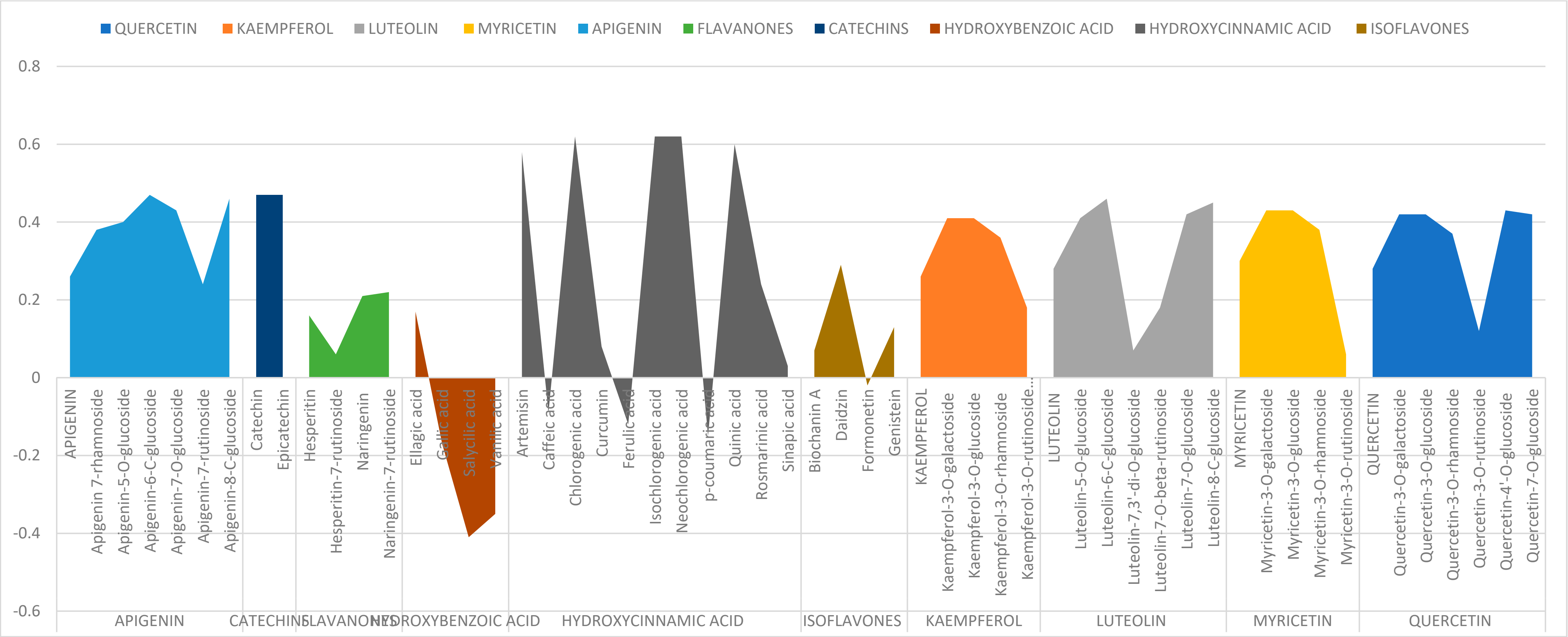

Figure S13. Plots of Nuclear receptor ligand scores.

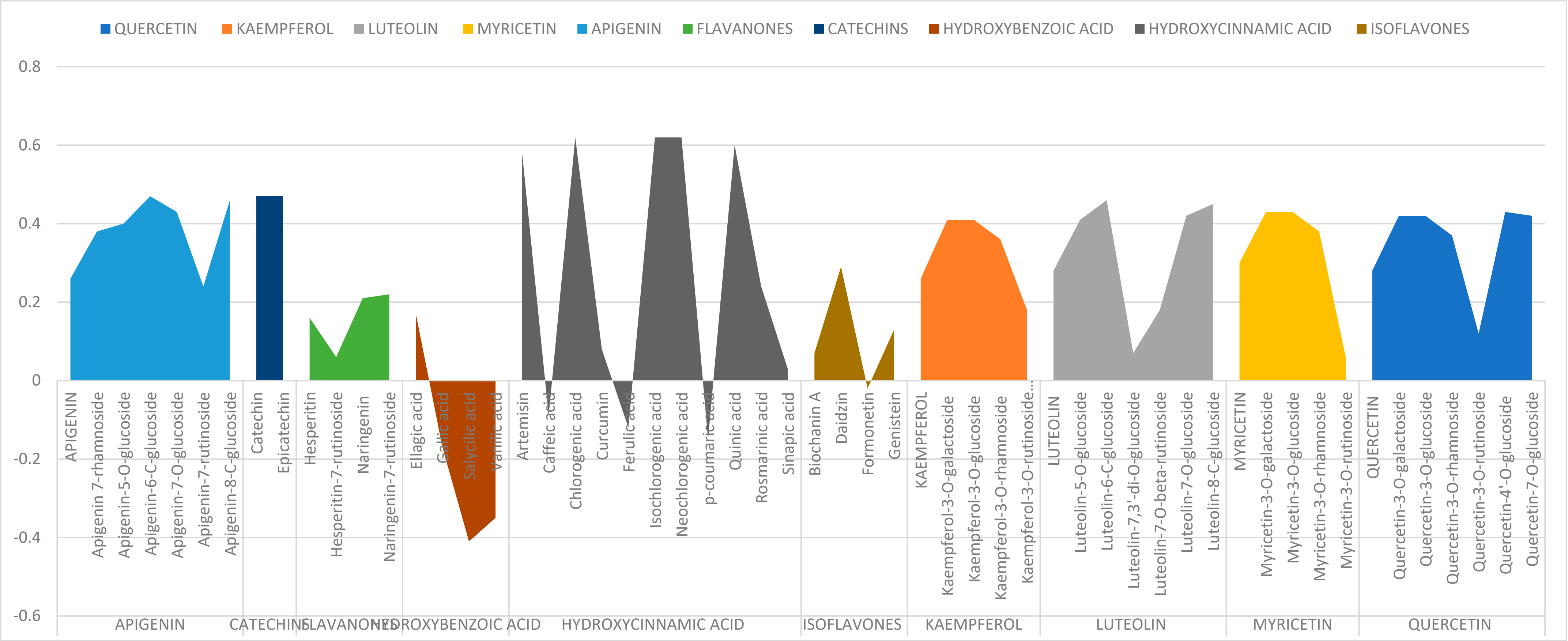

Figure S14. Plots of protease inhibitor scores.

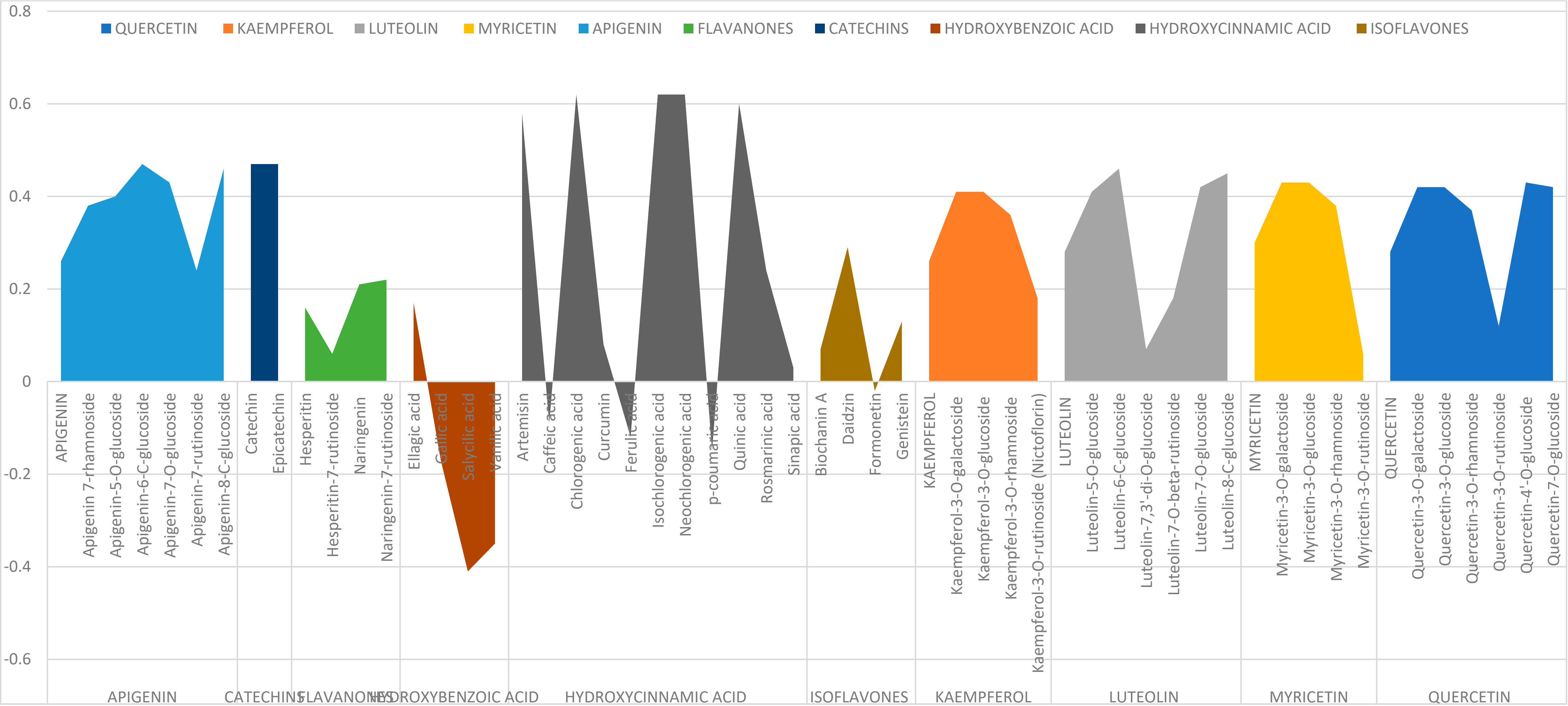

Figure S15. Plots of enzyme inhibitor scores.

Table S1. The content of the studied compounds in food products

Ccompound	Mean content (mg %)	Food products with a rich content in the studied plant compounds and/or the subclass they belong to (mg%), and the richest sources in the series in agreement with the specialized databases (http://phenol-explorer.eu/compounds/classification)
Apigenin	0 – 5	**parsley** (**215.50**), marjoram (4.4), oregano (3.5), sage (2.4), green celery hearts (19.1)
Apigenin glycosides	0 - 50	**common verbena (295), celery seed (111)**, common wheat (up to 46.82), Mexican oregano (28.33), black olive (8.18), globe artichoke (7.40), orange (5.53)
Luteolin	0 - 50	**Mexican oregano (56.33),** globe artichoke (42.10), thyme (39.50), sage (33.40)
Luteolin glycosides	0 – 50	**peppermint (1170), celery seed (up to 632), common verbena (495), Mexican oregano (297.67),** black olives (14.50), lemon verbena (10.60), globe artichoke (8.30),
Kaempferol	0 - 50	**capers (104.29),** cumin (38.60), cloves (23.80), caraway (16.40)
Kaempferol glycosides	0 - 50	**saffron (up to 255.32), capers (up to 165.76),** beans (up to 39.88), endive (up to 18.50), tea (up to 17.36), broccoli (16.60)
Quercetin	0 - 50	Mexican oregano (42), black elderberry (42), capers (32.82), cloves (28.40), cocoa/chocolate (25)
Quercetin glycosides	0 - 50	**capers (332.29), onion (up to 77.08),** olive (45.36), buckwheat (up to 36.14), black chokeberry (up to 46.46), asparagus (23.19), black tea (up to 19.68), spinach (19.17), red/black raspberry (up to 19), lingonberry (13.22), American cranberry (10.8), high-bush blueberry (8.99), lettuce (10.44), broccoli (6.50), beans (up to 6.30), plum (5.9)
Myricetin	0 - 5	red wine (0.83)
Myricetin derivates	0 - 5	highbush blueberry (12.21), black currant (3.14)
Naringenin	0 - 5	**Mexican oregano (372),** grapefruit (1.56)
Naringin	0 - 50	**rosemary (55.05),** grapefruit (up to 45.05),
Hesperidin	0 - 50	**peppermint (480,65), orange (up to 55.68),** lemon (up to 24.99), tangerine (36.11)
Genistein	0 - 10	soy tempe (10), tofu fermented (9.68)
Daidzin	0 - 100	**soy paste cheonggukang (85.85), soy flower (77.31)**
Formonetin	0 - 10	soybean roasted (6.16)
(+)-Catechin	0 - 10	**cocoa/chocolate (up to 107,75**), plum (up to 24.70), bean (up to 16.23), red wine (6.8), strawberry (6.36), peach (5.47), grape (5.46)
(-)-Epicatechin	0 - 10	**cocoa powder (up to 158.30), cocoa/chocolate (up to 70.36**), bean (up to 37.55), apple (up to 28.67), peach (7.97), tea (up to 7.93), grape (5.24), red raspberry (5.05)
Gallic acid	0 - 50	**chestnut (479.78), cloves (458.19),** chicory (up to 25.84), walnut liquor (15.15), sage/oregano (up to 5.25),
Ellagic acid	0 - 50	**chestnut (735.44)**, black berry (43.67), black raspberry (38), walnut (up to 28.50), cloud berry (15.30), pomegranate (up to 17.28),
Salicylic acid	0 -50	rosemary/thyme (up to 45), Salix bark (up to 11), brassica varieties (up to 7)
Vanillic acid	0 - 15	sweet basil (14), oregano (6), thyme (6.10), sage (up to 5.85)
p-coumaric acid	0 - 10	cloves (8.49), peanut (6.46), green olive (5.90), date (5.77), thyme/sage (4.95%), cloudberry (4.30%)
Ferulic acid	0 - 10	**hard wheat (72.21)**, common wheat (8.08), date (11.83), common wheat (8.08), marjoram/thyme/sage (5-7)
Feruloyl acid derivates	0 - 30	coffee (up to 30)
Diferulic acid derivates	0 - 15	Rye (14.58), hard wheat (11.02), maize (5.43) and turmeric (3.14% curcumin)
Caffeic acid	0 - 50	**black chokeberry (141,14),** lingonberry (6.34), plum (5.14), sage/ thyme/ oregano/ rosemary/ spearmint (up to 25), carraway/ cumin/ ginger/ nutmeg/ star anise (up to 25), sunflower oil (8.17)
Caffeoylquinic acid derivates	0 - 50	**plum (up to 118,59), coffee (up to 60),** cherries (up to 44.71), apples (5), broccoli (5.87)
Rosmarinic acid	0 - 500	**peppermint (up to 1734.49), spearmint (up to 900.67), thyme (up to 829), sage (up to 610.25), oregano (up to 599), rosemary (up to 987), sweet basil (up to 308)**
Sinapic acid	0 - 50	Green olives (44), black olives (10.8)
